# The ancient history of the structure of ribonuclease P and the early origins of Archaea

**DOI:** 10.1186/1471-2105-11-153

**Published:** 2010-03-24

**Authors:** Feng-Jie Sun, Gustavo Caetano-Anollés

**Affiliations:** 1 Evolutionary Bioinformatics Laboratory, Department of Crop Sciences, University of Illinois at Urbana-Champaign, Urbana, Illinois 61801, USA; 2 Laboratory of Molecular Epigenetics of the Ministry of Education, School of Life Sciences, Northeast Normal University, Changchun 130024, Jilin Province, PR China; 3 W.M. Keck Center for Comparative and Functional Genomics, Roy J. Carver Biotechnology Center, University of Illinois at Urbana-Champaign, Urbana, Illinois 61801, USA

## Abstract

**Background:**

Ribonuclease P is an ancient endonuclease that cleaves precursor tRNA and generally consists of a catalytic RNA subunit (RPR) and one or more proteins (RPPs). It represents an important macromolecular complex and model system that is universally distributed in life. Its putative origins have inspired fundamental hypotheses, including the proposal of an ancient RNA world.

**Results:**

To study the evolution of this complex, we constructed rooted phylogenetic trees of RPR molecules and substructures and estimated RPP age using a cladistic method that embeds structure directly into phylogenetic analysis. The general approach was used previously to study the evolution of tRNA, SINE RNA and 5S rRNA, the origins of metabolism, and the evolution and complexity of the protein world, and revealed here remarkable evolutionary patterns. Trees of molecules uncovered the tripartite nature of life and the early origin of archaeal RPRs. Trees of substructures showed molecules originated in stem P12 and were accessorized with a catalytic P1-P4 core structure before the first substructure was lost in Archaea. This core currently interacts with RPPs and ancient segments of the tRNA molecule. Finally, a census of protein domain structure in hundreds of genomes established RPPs appeared after the rise of metabolic enzymes at the onset of the protein world.

**Conclusions:**

The study provides a detailed account of the history and early diversification of a fundamental ribonucleoprotein and offers further evidence in support of the existence of a tripartite organismal world that originated by the segregation of archaeal lineages from an ancient community of primordial organisms.

## Background

With few exceptions [1], ribonuclease P (RNase P) is one of two universal ribozymes (the other is the ribosome) that are present in all living organisms. This ribonucleoprotein is generally composed of an RNA subunit, the RNase P RNA (RPR), and one or more protein subunits, the RNase P proteins (RPPs) [2]. RNase P functions as a phosphodiesterase carrying out the 5' endonucleolytic cleavage of transfer RNA (tRNA) precursor transcripts (pre-tRNA) to form mature functional tRNAs [3-5]. Regions of the RPR that contribute to the recognition of the substrate cleavage sites [the tRNA pseudouridine (TΨC) loop and CCA tail] are well studied. Remarkably, the catalytic function can be conducted by the RNA subunit independently of protein subunits, indicating that the biological activity resides in the RPR [6-8].

The ubiquitous distribution of RPR molecules in life suggests that a primordial RPR form was already present before the diversification of the three superkingdoms of life, Archaea, Bacteria, and Eukarya [9]. Furthermore, the RPR is also the catalytic subunit in all three superkingdoms [10]. Bacterial RPRs have been divided into two independently folding domains, the catalytic (C) domain involved in substrate cleavage and the specificity (S) domain involved in substrate binding [11,12]. The S domain is composed of stem P7 and stems distal to P7 while the rest of the molecule delimits the C domain. The C domain contains the entire active site and binds the acceptor stem/5'-leader and the ACCA sequence at the 3' end (by a Watson-Crick base-pairing mechanism) of pre-tRNA, cleaving the leader sequence in the presence of bacterial RPP cofactors [13,14]. The S domain binds the TΨC stem-loop region of pre-tRNA and confers substrate specificity. RPR can be divided into five universally distinct conserved regions (CR I to V) that are distal to each other in the primary sequence and define the universally conserved core structure [15]. The S domain comprises CR II and III and the C domain comprises CR I, IV, and V [16]. While components of the tertiary fold are overwhelmingly helical it is interesting that both domains have many nonhelical parts: CR II and III form two interleaving T-loop motifs whereas CR I, IV, and V are part of loops and turns.

Altman and Kirsebom [17] proposed that an earlier RPR form that lacked the S domain might have existed in the RNA world, because this domain was not needed for the binding of the substrate. The C domain was therefore more ancient than the S domain. Remarkably, modification of the S domain of *Bacillus subtilis *indicates substrate specificity can be altered without changing the basic cleavage reaction [18]. In fact, a "minimal" RPR, the smallest molecule needed to carry out the hydrolysis reaction, has been defined [19]. This minimal RPR contains molecular components from both the S and C domains. Interestingly, consensus RNase P structures show that the C domain is more conserved than the more variable S domain in all three superkingdoms [19-22]. However, the C domain by itself is either non-functional in the absence of cognate RPPs or has greatly decreased catalytic activity compared to the wild type [23-25]. Furthermore, the S domain appears to facilitate substrate recognition and binding in the ribozyme reaction and the S domain alone can bind pre-tRNA directly [26]. All these observations suggest the C domain is indeed ancestral and that the S domain plays an accessory but important role during the cleavage of precursor tRNA. Despite the relevance of these results, the evolutionary history of the molecular components of the two structural folding domains remains elusive.

**Figure 1:**
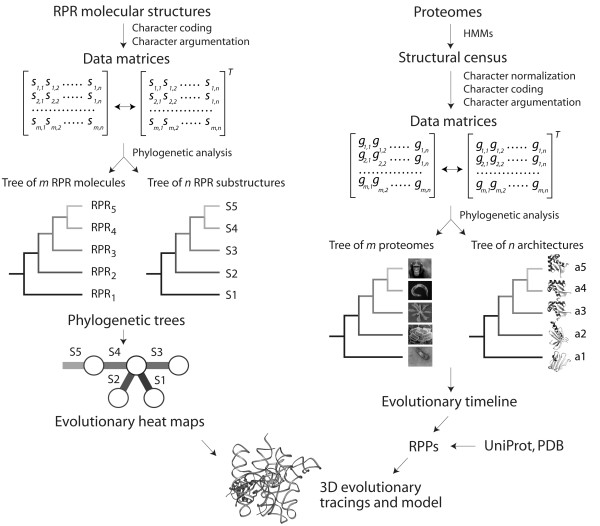
**General methodological approach**. The flow diagram in the left describes the phylogenetic reconstruction of trees of molecules and substructures. The structure of RPR molecules can be deconstructed into substructures, such as coaxial stem tracts and unpaired regions, that can be studied using features (characters) that describe their geometry and shape (e.g., length of stems or unpaired regions). These structural characters are coded and assigned "character states" according to an evolutionary model that polarizes character transformation towards an increase in molecular order (character argumentation). Coded characters (*s*) are arranged in data matrices, which can be transposed and subjected to cladistic analyses to generate intrinsically rooted phylogenetic trees of either molecules or substructures. The trees are then used to generate evolutionary heat maps of secondary structure that color secondary structures or 3D structural models with molecular ancestries. The flow diagram in the right shows the reconstruction of trees of proteomes and trees of protein architectures. A census of domain structures in proteomes of hundreds of completely sequenced organisms is used to compose a data matrix and its transposed matrix, which are then used to build phylogenomic trees describing the evolution of individual architectures and entire molecular repertoires, respectively. Elements of the matrix (*g*) represent genomic abundances of architectures (at FSF level of hierarchical classification of structure) in proteomes. Ancestries derived from the tree of architectures can be "painted" onto 3D models of the RNase P molecular complex. Evolutionary information from RNA and protein structures is finally combined to generate a model of structural evolution.

Here we study the evolution of the RNase P complex with a well-established phylogenetic method that reconstructs evolutionary history directly from structure [27]. This cladistic approach produces intrinsically rooted trees that "embed structure and function directly into phylogenetic analysis" [28]. The method has been applied widely to study the evolution of structure in rRNA [27,29], tRNA [30-32], SINE RNA [33], and other molecules [34], and has also been extended to the evolutionary study of protein domains at fold and fold superfamily (FSF) levels of structural complexity [35-38]. Two kinds of trees are generated in studies of RNA evolution, 'trees of molecules' that describe the evolution of molecular lineages, and 'trees of molecular substructures' that describe the evolution of structural components of the molecules. Using this methodology, we here study the history of the structure of the RNA subunit, establishing how the shape of the RPR molecule and its structural domains changed in evolution (Figure 1). This information was then coupled with an evolutionary analysis of RPP domain structures at FSF level using previously developed methods of phylogenomic reconstruction [38]. Finally, the evolutionary tracing of phylogenetic information in crystallographic models (heat maps) help clarify how the history of the ribonucleoprotein relates to the discovery of function and the establishment of RNA-protein interactions.

## Results and Discussion

### A cladistic strategy to study the evolution of molecular structure

We illustrate our cladistic approach with a flow diagram that describes how we study RNA and proteins (Figure 1). When analyzing RNA molecules, we first deconstruct RNA secondary structure into substructures, very much as nucleic acid sequences are deconstructed into nucleotide sites for the purpose of phylogenetic analysis. RPR crystal structures show that a substantial portion of the molecule is helical or approximately helical, a feature that RPR shares with rRNA and other functional RNA. For example, the ribosomal ensemble can be effectively considered an arrangement of ~200 helical segments in three-dimensional (3D) space [39]. In these molecules, the accretion of disparate helical segments contributes to the aggrandizement of ribosomal structure, which is ultimately responsible of making up crucial functional centers [40]. Similarly, the RPR molecule can be considered an arrangement of ~26 helical substructures (P1, P2, etc.), some of which are missing in particular molecular lineages (see below). Since crystallographic models and comparative sequence analysis support the existence and homology of these substructures, attributes describing structural features of these substructures (e.g., their length) can be used as phylogenetic characters to build either trees of molecules or trees of substructures (Figure 1). The attributes of these substructures are therefore analogous to the presence of a nucleotide at a particular site in a nucleic acid sequence in traditional phylogenetic reconstructions, with the caveat that it would be indeed challenging to build trees of sites (instead of trees of sequences) directly from sequence.

**Figure 2:**
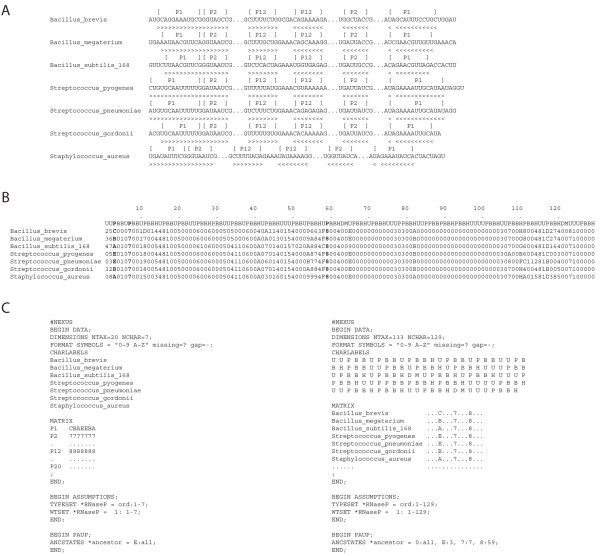
**Fundamental steps in phylogenetic reconstruction**. (A) Seven bacterial RPR sequences are randomly selected from the 133 accessions used in the present study and segments of the sequences between "..." are used to illustrate the procedure. Brackets above the sequences delimit three helical regions (P1, P2, and P12). Paired bases are marked using ">" and "<" under the sequences. (B) Complete data matrix describing the length of a total of 129 substructures derived from the annotated sequences. These substructures and their character states are described in Additional file 1: Tables S1 and S2. Substructures P1, P2, and P12 are highlighted in bold. (C) The input files in NEXUS format used to generate phylogenetic trees of substructures (left) or molecules (right) using PAUP*. The files contain the data matrices with description of characters and taxa, phylogenetic assumptions, and the hypothetical ancestral states defined by command "ANCSTATES" under the "DATA", "ASSUMPTIONS", and "PAUP" blocks. Only helical substructural characters for P1, P2 and P12 (left) and seven RPR molecules (right) are listed as taxa in the matrices.

We illustrate the analysis in more detail with a concrete example of how we build trees of molecules and trees of substructures (Figure 2). We first generate primary RPR sequence alignments that take into consideration the secondary structure of the molecules. We then score the lengths of segments that form base pairs or remain unpaired, traversing from the 5' to the 3' end of the molecules that are examined. In this process we assign substructures to stems, bulges, hairpins, and other unpaired segments as we encounter these in the secondary structure. This defines a data matrix with for example columns describing substructures and rows describing the RPR molecules. These matrices can be partitioned into matrices of substructures types (e.g. stem substructures) and encoded in the NEXUS format (as is or transposed), which are then used as input text file for equally weighted unconstrained maximum parsimony (MP) analysis. In the file, the first column represents a phylogenetic character and individual numerical values character states. Rows are phylogenetic taxa. The input files generate rooted trees of molecules or trees of substructures depending on what is considered taxa (see Figure 1). The NEXUS file also defines the character states of a hypothetical ancestor (under the 'ANCSTATES' command) and this determines the preferred direction (polarity) of character state change. This hypothetical ancestor in our example has the maximal character state for each character and is included in the search for optimal rooted trees with the sole purpose of polarizing character state transformation. No external hypotheses in the form of outgroups (e.g. ancestral molecules or ancestral substructures) are needed to root the trees. The external hypotheses are replaced by the more axiomatic polarization assumption of our evolutionary model. For more detailed descriptions of the method and model, see Materials and Methods and for example [29-33,41].

With proteins, we use hidden Markov models (HMMs) of structural recognition to survey protein sequences in genomes, decomposing proteins into protein domains at FSF level (Figure 1). The survey establishes the number of copies of a domain that exist in the proteome of an organism that has been fully sequenced, and these numbers are used as character states when constructing data matrices, with columns and rows representing proteomes or domain architectures. As with RNA, matrices can be transposed to construct trees of proteomes or trees of architectures. Trees of architectures define the age of each individual domain (reviewed in [38]), and this information can be used to establish the relative and absolute age of the RPPs that are present in the RNase P complex.

### Phylogenetic utility of RPR structure and the early origin of Archaea

**Figure 3:**
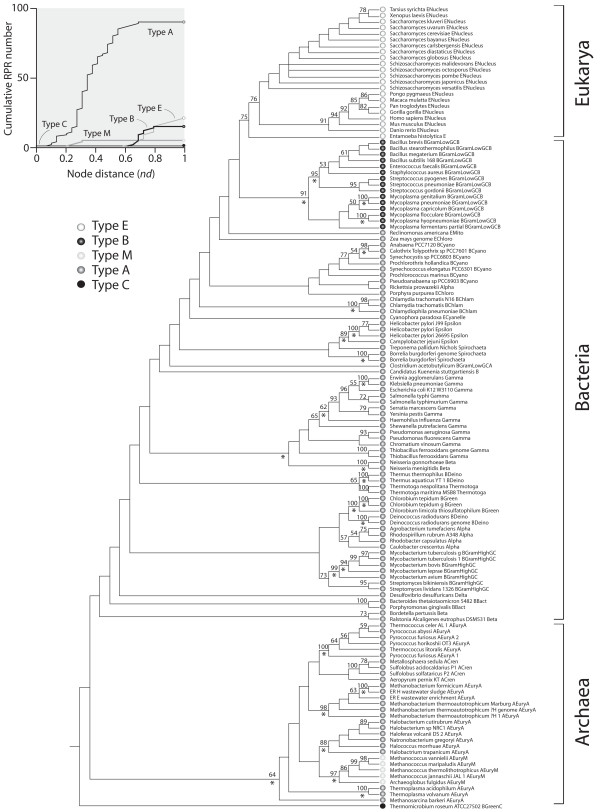
**Evolution of the structure and sequence of RPR molecules**. Phylogenetic maximum parsimony analyses of combined structure and sequence data in 133 RPRs resulted in 6 minimal length trees, each of 14,359 steps (CI = 0.248 and 0.240, with and without uninformative characters, respectively; RI = 0.712; RC = 0.176; g_1 _= -0.263). The figure shows a strict consensus of these trees, which is well resolved, and lists bootstrap support (BS) >50% for individual nodes. Asterisks below branches indicate groups also recovered by separate analyses of sequence or structure data. Symbols in terminal leaves describe the structural type of the RPR molecules. The inset shows a cumulative frequency distribution plot describing the accumulation of molecular types along a timeline defined by the distance (*nd*) in nodes from the ancestral substructure at the base of the tree, on a relative scale.

Recent studies have indicated that RPR molecules are suitable for phylogenetic analysis of closely related bacterial taxa and have potential as a tool for species discrimination [42]. One distinctive feature is that there is only one copy of the encoding gene in a genome making it more refractory to inter-specific lateral gene transfer. Moreover, RPRs from Archaea, Bacteria, and Eukarya are easily distinguished from each other, serving as good and reliable molecular markers for systematic phylogeny. Sequence diversity expresses even if the structure and associated catalytic function of the molecules remain unchanged [43]. This feature becomes extremely useful when exploring deep evolutionary relationships, especially in cases where sequences are too variable or when molecules that are compared are distantly related (e.g., RPR and a variant that participates in rRNA processing, RNase MRP RNA) [44]. In this regard, Collins et al. [45] demonstrated that phylogenetically informative characters are indeed embedded in the secondary structure of RPR molecules and that these can be used to uncover the tripartite nature of life heralded by the Woese school. In the present study, we reconstructed phylogenetic trees describing the evolution of 133 RPRs using information in sequence and structure (Table 1). These phylogenies were generally well resolved and clustered molecules belonging to the three superkingdoms, with Archaea and Eukarya generally appearing unified in single (monophyletic) groups (Table 2). Since structural phylogenetic characters provide a direction to evolutionary change without the need of outgroups or external hypotheses of relationship [27,44], their inclusion established patterns of origin and was therefore particularly advantageous. Figure 3 describes a rooted tree generated using the total evidence approach from both structure and sequence data. With the exception of a single bacterial molecule that appeared at the base of the tree and harbored a unique structural type (see discussion below), archaeal RPRs were ancient while eukaryal RPRs were derived. This rooting of the tree of molecules suggests an early diversification of Archaea. The result is particularly remarkable, especially because it is congruently supported by phylogenetic analyses of tRNA paralogues [46-49], the structure of tRNA [31] and 5S rRNA [41], and phylogenomic studies of domain structure [37] and domain organization in proteins [35,36].

**Table 1:** Sequence and structural features of the RPR molecules analyzed (*)

Sequence characteristics	Archaea	Bacteria	Eukarya	Combined
Number of molecules	30	77	26	133
Nucleotide sequence length	229-475	315-485	273-383	233-486
No. of aligned positions	887 (129)	1040 (129)	404 (129)	692 (129)
No. of aligned positions constant	462 (60)	279 (24)	18 (45)	40 (9)
No. of aligned positions parsimony-informative	308 (54)	578 (98)	358 (65)	616 (110)
No. of aligned positions autapomorphic	117 (15)	183 (7)	28 (19)	36 (10)
Maximum pairwise sequence divergence (%)	62.2	63.1	75.6	80.8

(*) - Information on structural characters is given in parentheses. Source of eukaryal molecules: nucleus (22), mitochondia (1), chloroplast (2), and cyanelle (1).

**Table 2:** Statistics of trees of RPR molecules (*)

Matrix	No. of trees	Tree length	CI	RI	RC	g_1_	Archaea	Bacteria	Eukarya
Archaea									
Structure (30/129)	8	1,003	0.49/0.46	0.70	0.34	-0.75	—	—	—
Sequence (30/887)	5	1,523	0.81/0.71	0.84	0.68	-0.54	—	—	—
Combined (30/1,016)	2	3,451	0.63/0.53	0.67	0.42	-0.62	—	—	—

Bacteria									
Structure (81/129)	162	2,453	0.30/0.30	0.75	0.23	-0.55	—	—	—
Sequence (81/1,040)	7	5,107	0.38/0.31	0.61	0.23	-0.51	—	—	—
Combined (81/1,169)	4	8,799	0.39/0.34	0.65	0.26	-0.52	—	—	—

Eukarya									
Structure (22/129)	1	1,090	0.55/0.54	0.80	0.44	-0.60	—	—	—
Sequence (22/404)	32	1,531	0.60/0.59	0.77	0.47	-0.58	—	—	—
Combined (22/533)	8	3,042	0.63/0.61	0.78	0.49	-0.58	—	—	—

All superkingdoms									
Structure (133/129)	>10,000	4,260	0.25/0.25	0.81	0.20	-0.24	+/+	-/-	+/+
Sequence (133/692)	>10,000	8,564	0.22/0.21	0.64	0.15	-0.30	+/+	-/-	-/-
Combined (133/821)	6	14,359	0.25/0.24	0.72	0.18	-0.26	+/+	-/-	+/+

Structural characters									
Stabilizing (133/26)	>10,000	1,476	0.23	0.81	0.19	-0.20	-	-	-
De-stabilizing (133/77)	>10,000	2,210	0.29/0.28	0.84	0.24	-0.25	+	-	+

Folding domains									
S domain (133/42)	>10,000	1,478	0.32	0.85	0.267	-0.21	-	-	-
C domain (133/87)	>10,000	2,342	0.25/0.25	0.83	0.210	-0.24	+	-	+

(*) - Trees were derived from data matrices partitioned according to superkingdom, or structural characters of RPR folding domains, and from complete data matrices of sequence, structure, or combined sequence and structure. The number of taxa/characters analyzed is shown in parentheses. Symbols "+" and "-" are used to indicate the existence of monophyletic or non-monophyletic groupings of RPRs, respectively (data of the trees derived from MP and NJ analyses are given in tandem). CI, consistency index (with and without uninformative characters are given in tandem); RI, retention index; RC, rescaled consistency index; symbol "-" indicates non-applicable data.

Detailed phylogenetic patterns were maintained when data were partitioned according to superkingdoms or according to sequence, structure, or structural domain (Table 2). As previously reported for other RNA molecules [27,29,30,34,45,50,51], trees derived from structure were largely congruent with those derived from sequence, both from combined data or matrices partitioned according to superkingdoms, with the incongruent nodes being weakly supported by bootstrap values (<50%) and generally basal in the trees. Congruence was also observed when comparing trees generated by neighbor-joining (NJ) and MP analyses. In terms of superkingdoms, only trees reconstructed from the S domain or from stabilizing structural characters failed to reveal the monophyly of both Archaea and Eukarya. Trees generated from the C domain (using both stabilizing and de-stabilizing structural characters) were better resolved than those derived from the S domain (Additional file 1: Figures S1 and S2). Similarly, trees derived from de-stabilizing characters were better resolved than those obtained from stabilizing characters. Interestingly, trees derived from helical structure conserved in all three superkingdoms were largely unresolved (Additional file 1: Figures S3 and S4). Overall results strongly support the generally accepted concept that ancient substructures that are more stable and are universal have less power to resolve phylogenetic relationships of lineages than derived substructures that are less stable, are lineage specific, or are believed part of derived structural domains.

In Archaea, the monophyly of Crenarchaeota was recovered by the combined analysis of structure and sequence data (Figure 3) but not by separate analyses of either structure or sequence data alone. The monophyly of Euryarchaeota was not revealed in any analysis. This result agrees with whole-genome studies that have questioned the monophyly of these two groups [52,53]. Furthermore, the monophyly of Euryarchaeota and Crenarchaeota is based on 16S rRNA, the most popular gene for evolutionary studies. However, it is now becoming apparent that there is only partial agreement between the 16S rRNA universal tree and phylogenies derived from proteins or genomic complements [54]. In Bacteria, major bacterial groups were clearly identified but branching patterns were mostly unresolved. This is an expected result, especially because bacterial phylogeny has not been convincingly reconstructed and the issue of the branching order of major bacterial lineages remains contentious. For example, phylogenetic analyses of a large dataset of all available bacterial RPR sequences resulted in unstable tree topologies [55]. Observations are therefore consistent with RPRs being of limited phylogenetic use in Bacteria: relationships among phyla were largely unresolved while relationships within phyla were well-resolved and comparable to relationships within superkingdoms Archaea and Eukarya.

### Evolution of RPR types

An accurate model for RPR structure is a prerequisite for understanding the mechanism of substrate recognition (S domain), catalytic activity (C domain), and evolution of the ribozyme, and is supported by a significant body of evidence from biochemical, photochemical, molecular, crystallographic, and phylogenetic comparative studies. For example, refined secondary structure models have been inferred by identifying concerted changes (covariation) in the nucleotide sequence of RPRs that share common ancestry and function and have been generally confirmed by biochemical and crystallographic evidence [56-59]. In these analyses, the eukaryal secondary structure is not sufficiently resolved [60], and although there are no high-resolution structures available for archaeal and eukaryal RPRs, the identification of more than 50 sequences from each superkingdom allowed considerable refinement of secondary structure models [22,61,62]. Five general types of RPR structures are recognized in molecules belonging to the three superkingdoms (Table 3). Covariation analyses of a comprehensive set of bacterial RPR sequences established a well-defined secondary structure, identified tertiary interactions, and classified RPRs into two major classes with distinct secondary structures [3]. The common ancestral-type or type A structure, represented by *Escherichia coli*, is found in most bacterial and archaeal organisms. The *Bacillus*-type or type B, represented by *Bacillus subtilis*, is found only in low-GC Gram-positive bacteria [63]. Through a process of convergent molecular evolution, most of the unusual structural elements of type B RPRs evolved independently in *Thermomicrobium*, a member of the green non-sulfur bacteria, to form type C RPRs (lacking stems P13 and P14 but containing P10.1; Table 3) [63,64]. In Archaea, the *Methanococcus *and *Archaeoglobus fulgidus *RPRs form a unique derived structure class, type M, with an apparently less complex structure (lacking P8) in comparison to the ancestral type A structure [61,64]. Finally, eukaryal RPRs generally lack a convincing secondary structure model and are distinct from those of Bacteria and Archaea. They are defined as type E.

**Table 3:** Taxonomic distributions of helical substructures in RPR molecules (*)

Helical substructures	*nd *values	Archaea		Bacteria			Eukarya
		
		Type A	Type M	Type A	Type B	Type C	Type E
P12	0.00	+	+	+	+	+	+
P1	0.05	+	+	+	+	+	+
P3	0.10	+	+	+	+	+	+
P4	0.15	+	+	+	+	+	+
P2	0.20	+	+	+	+	+	+
P10-11	0.25	+	+	+	+	+	+
P9	0.30	+	+	+	+	+	+
P8	0.35	+	-	+	+	+	+
P7	0.40	+	+	+	+	+	+
P5	0.45	+	+	+	+	+	+
P15	0.50	+	+	+	+	+	+
P6	0.55	+	-	+	-	+	-
P16	0.60	+	-	+	-	+	+
P17	0.65	+	-	+	-	+	-
P19	0.75	+	-	+	+	-	+
P13	0.75	-	-	+	-	-	-
P18	0.80	-	-	+	+	+	-
P16.1	0.80	+	-	+	-	-	-
P14	0.80	-	-	+	-	-	-
P16-17	0.85	-	-	+	-	-	-
P20	0.90	-	-	+	+	-	-
P15-16	0.90	+	-	-	-	-	-
P16.2	0.90	+	-	-	-	-	-
P5.1	0.95	-	-	-	+	-	-
P10.1	1.00	-	-	-	+	+	+
P15.1	1.00	-	-	-	+	+	-

(*) - Substructures sampled in the present study are ordered by *nd *values. "+" and "-" indicate the presence or absence of the helical substructures, respectively.

Trees of molecules dissected the evolutionary history of the different types of RPR structures and their evolutionary origin (Figure 3). Mapping of the various types of RPRs on the trees revealed patterns of origin and evolution of structural design (Figure 3). Type A molecules in Archaea and Bacteria were clearly ancestral compared to type B molecules, while type M structures of methanogenic archaeal species appeared quite early in the monophyletic archaeal group. Eukaryal type E molecules were the most derived in the tree. It is generally acknowledged that type A is the most ancient folding structure in RPRs [65] and that the ancestral type A form underwent substantial innovative change in the common specific ancestry of the eukaryal and archaeal lineages. This change can be visualized in the make-up of the RNase P complex. For example, enzymes in both Archaea and Eukarya contain more proteins than those in Bacteria, suggesting that archaeal and eukaryal RPRs have coevolved to display a greater dependence on their cognate proteins. The eukaryal RPRs are weaker catalysts than their bacterial counterparts, supporting the notion that RPPs play important functional roles, assisting for example in RNA folding, substrate binding, and/or catalysis. Although archaeal RPRs are composed of an RNA subunit similar to bacterial RPRs [61,66], the multiple protein subunits are similar to those in the eukaryotic nucleus [65]. More importantly, a few bacterial RPR structural elements that are essential for substrate binding, catalysis, and global stability were either never acquired or lost during evolution of the archaeal and eukaryal RPRs, accounting for their lower stability/activity in the absence of cognate RPPs [3,67,68]. Furthermore, the archaeal and eukaryal RPRs are clearly missing sequence/structure elements present in bacterial RPRs that are either important for tertiary contacts or for direct interactions with the substrate (Table 3). For example, the L15 loop (of the P15 substructure) that establishes base-pairing interactions with the CCA sequence at the 3' end of pre-tRNAs is also absent in all eukaryal and some archaeal RPRs. These observations suggest that archaeal and eukaryal RPRs underwent reductive evolutionary tendencies similar to those seen in the very ancient components of proteomic repertoires [37].

Interestingly, the type C RPR structure from *Thermomicrobium roseum *was placed at the base of trees of molecules (Figure 3). Type C and type B RPRs are not phylogenetically related at sequence level and present major structural differences, including the presence of P6, P16, and P17 in type C and their replacement with P5.1 in type B (Table 3). However, they also share structural features (e.g., loss of P13 and P14 and acquisition of P10.1) resulting from convergent molecular evolution events [63].

### Evolution of RPR structure

**Figure 4:**
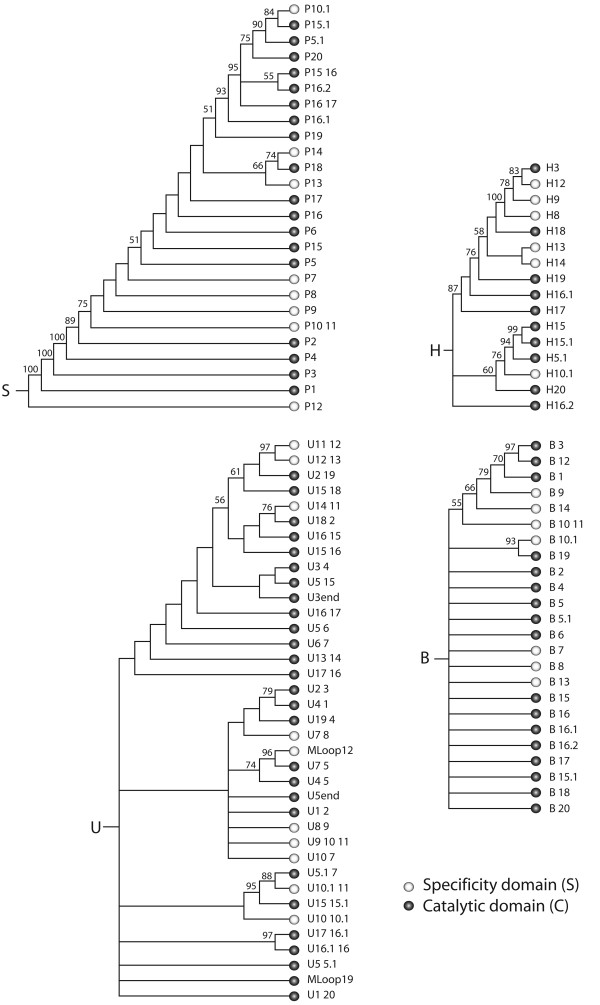
**Evolution of RPR structure**. Trees of molecular substructures reconstructed from geometrical characters of RPR structure. Trees of substructures describe the evolution of stems (S) (12,121 steps; CI = 0.790, RI = 0.772; RC = 0.610; g_1 _= -0.758), bulges and internal loops (B) (1,168 steps; CI = 0.775 and 0.771, with and without uninformative characters, respectively; RI = 0.546, RC = 0.423; g_1 _= -1.884), hairpin loops (H) (1,949 steps; CI = 0.591, RI = 0.765, RC = 0.452; g_1 _= -0.556), and unpaired segments that include external segments (free ends) and multiloop regions (U) (5,277 steps; CI = 0.374, RI = 0.634 RC = 0.237; g_1 _= -0.719). BS >50% are shown for individual nodes. Symbols in terminal leaves describe substructures in domains.

**Figure 5:**
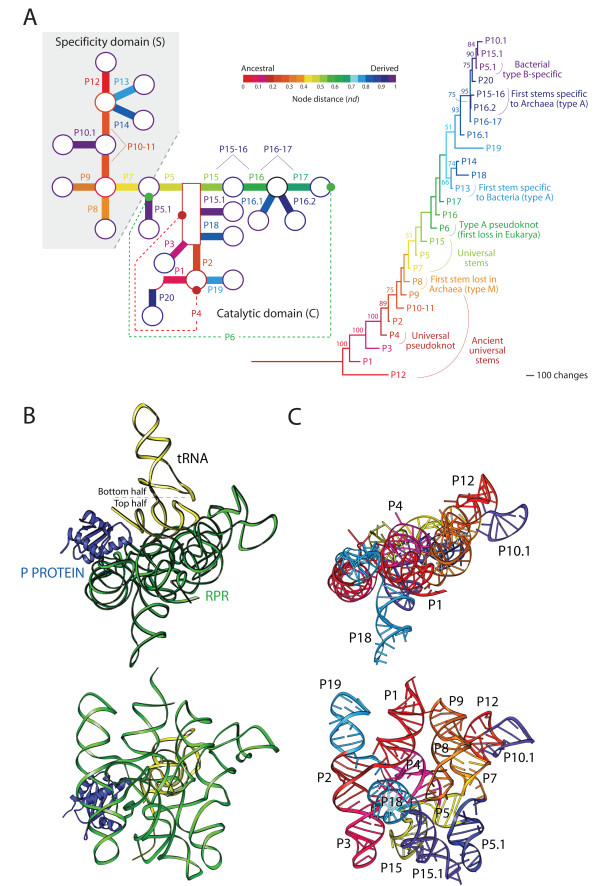
**The evolutionary history of the structure of RNase P**. (A) Trees of molecular substructures were reconstructed from characters describing the geometry of RPR structure. The tree of stem substructures of Figure 3 is shown with branches colored according to node distance (*nd*). The same ancestry color scale was used to paint a schematic drawing of the secondary structure of a consensus RPR, with stems drawn as thick lines and loops as circles. This evolutionary heat map describes the relative addition of fundamental structural components to the evolving molecule. (B) Top and lateral views of the RNase P ternary complex, using a model described in ribbons format based on Buck et al. [69]. The interaction of RPR with tRNA and RPP molecules is shown. (C) Same top and lateral views of the RPR molecule with the relative ages of RPR substructures mapped onto the 3D model. Note how the RPP interacts with the ancient P2 and P3 stems, and laterally with the P4 pseudoknot, and how the top half of tRNA makes crucial contacts with the base of the most ancient substructure, the P12 stem. The RPR and RPP structure is from *Bacillus *[58,70].

Phylogenetic trees of RPR substructures (and associated evolutionary heat maps) provide a chronology that establishes which parts of the molecule are ancestral and which parts are derived. We generated trees describing the evolution of helical stems, hairpins, bulges, and unpaired regions (Figure 4; see Table 4 for tree statistics). Since RNA structures are defined by a frustrated conformational interplay of stems and loops, the tree of stem substructures (revisited in Figure 5) constitutes the fundamental scaffold of structural evolution of the entire molecule. Trees of stems derived from the complete dataset and from datasets partitioned according to the C and S structural domains revealed concordant topologies (Additional file 1: Figure S5). The analysis of other substructures was less informative but complemented the original evolutionary patterns derived from the stem scaffold (Figure 4). For example, the tree of unpaired regions showed the 5'-terminal free end of the molecule (U5end) was more ancient than the 3' end (U3end), a pattern that was recovered independently in the analysis of both tRNA and 5S rRNA molecules [30,41].

**Table 4:** Statistics of trees of RPR substructures (*)

Matrix	No. of trees	Tree length	CI	RI	RC	g_1_
S domain						
stems	1	10,118	0.95	0.80	0.76	-2.44
hairpins	1	1,182	0.84	0.80	0.67	-0.87
bulges	2	785	0.89/0.88	0.58	0.51	-1.04
unpaired	1	2,465	0.77/0.77	0.75	0.58	-2.34
C domain						
stems	2	11,293	0.85	0.77	0.65	-0.65
hairpins	1	1,289	0.67	0.65	0.44	-0.76
bulges	12	501	0.86/0.79	0.53	0.46	-2.72
unpaired	9	3,574	0.43	0.61	0.26	-0.52
Combined						
stems	2	12,121	0.79	0.77	0.61	-0.76
hairpins	1	1,949	0.59	0.77	0.45	-0.56
bulges	109	1,168	0.78/0.77	0.55	0.42	-1.88
unpaired	8	5,277	0.37	0.63	0.24	-0.72

(*) - Trees were derived from data matrices partitioned according to structural domains and complete data matrices. CI, consistency index (with and without uninformative characters are given in tandem); RI, retention index; RC, rescaled consistency index.

The most ancient substructure in the tree was P12, a terminal stem of the S domain (Figure 5A). This substructure was immediately followed by four helical segments of the C domain, P1, P3, P4 and P2, in that order, one of which (P4) represents the universal pseudoknot structure of the complex. Substructures P1 to P4 define an important molecular feature that is revealed on the tree as a paraphyletic basal group. This helix structure contains the RNase P active site [58,59,69-71], the catalytic center that cleaves the pre-tRNA sequence. Furthermore, substructures P1 to P4 are part of the conserved minimal core defined by Siegel et al. [19], which is shared by organisms in all three superkingdoms. The other shared substructure in this core is helix P10-11, another substructural component of the S domain, which appears in the tree immediately after the P1-P4 core structure. However, this substructure sustains considerable sequence variation, particularly among eukaryotic RPRs. Interestingly, the most conserved nucleotides in the RPR sequence are concentrated in the core structure (centered in P4) and in the large loop between P11 and P12 [72].

The taxonomic distribution of evolving stem substructures revealed that, with an exception in the P8 stem that was lost in type M archaeal molecules, the first 10 most ancient structures were universally present in all RPR molecules (Table 3). Note however that P12 is absent in *Mycoplasma fermentans *and Thermoproteaceae, species with highly reduced genomes [19,72]. The next structure to evolve was a pseudoknot (P6) that is typical of type A molecules in Archaea and Bacteria, but not present in Eukarya. This stem was followed by a number of structures generally shared by RPRs in one or more superkingdoms, with structures specific to bacterial type A RPRs evolving first, followed by structures specific to archaeal type A molecules, and then structures specific to bacterial type B RPRs (see summary of patterns in Figure 4A). These patterns define the possible emergence of superkingdoms and match the rooted topology of the tree of molecules derived from combined sequence and structure datasets, which suggests the early diversification of Archaea (Figure 3).

With the exception of stem P12, the C domain was in general more ancient than the S domain on all of the trees of stem substructures analyzed. The ancestral nature of the C domain was also revealed in trees of other substructures (Additional file 1: Figure S6). Overall, the highly conserved P1-P4 core structure was primordial in the C domain when compared to many other helical structures that were added later in evolution to both structural domains. Consequently, our phylogenetic constructs provide an additional and strong line of evidence in support of the ancestral nature of the C domain [17]. Note that exclusion of stem P12 and other basal substructures from the analysis did not alter the topology of the trees, supporting the robustness of our phylogenetic hypotheses (data not shown).

The catalytic core represents the set of four ancestral elements (P1 to P4) in the RPR molecule (Figure 5) and the only four conserved stems of the C domain in the universal consensus minimum structure of RPR [3]. It is therefore particularly noteworthy that these ancestral and conserved substructures interact with the ancient top domain of the pre-tRNA as it cleaves its 5' end sequence (Figure 4B). This top half of the pre-tRNA molecule is composed of the TΨC and acceptor arms. Previous phylogenetic studies indicate that the top domain of tRNA predates evolutionarily the bottom domain composed of the dihydrouridine (DHU) and anticodon arms [30] supporting the ancestrality of this part of the molecule [73]. Consequently, the relative age of molecular contacts suggests the co-evolution of the top domain of tRNA (the substrate) and the C domain of the RPR (the catalyst). Given the supporting (instead of catalytic) function carried out by the S domain, the ancestrality of the very ancient P12 substructure is clearly of evolutionary significance (see discussion below).

### Early origins of RNA-protein interactions in the catalytic complex

It is critical that we examine RNA-protein interactions in the RNase P complex, given that proteins and catalytic RNA are both required for enzyme activity *in vivo*. In addition to pre-tRNAs, there are a few other substrates for RNase P *in vivo*, such as pre-4.5S RNA, pre-tmRNA, a few mRNAs, and riboswitches [74-78]. To date, it is generally realized that RPR-RPP interactions serve to stabilize the structure of the complex, enhancing substrate recognition and affinity for substrates and metal ions [69,79-82].

Bacterial RNase P studies show that type B and ancestral type A molecules can interchangeably activate RNA catalytic functions [69,71,82,83] at both protein and RNA levels, indicating that the RPP recognizes a region of structure that is conserved between the two classes of bacterial RNase P enzymes [83]. The crystal structures of type A (*Thermotoga maritima*) and type B (*Bacullus stearothermophilus*) RPRs [16,58,59] revealed similar features in the catalytic cores of both RNAs, including the coaxial stacks P1/P4/P5, P2/P3, and P8/P9. Specifically, the metal binding loop and N-terminus of the RPP are near the P3 stem-loop of the RPR. Additionally, the conserved RNR motif is close to helix P4, which is necessary for positioning divalent metal ions required for catalysis, and is the putative active site of the holoenzyme [13,84,85]. These studies support the notion that the RPP binds a conserved area of the RPR, stabilizing the local RNA structure, as well as stabilizing the RPR contacts with the pre-tRNA substrates [69,82,86].

As discussed previously, our results support the hypothesis that the C domain is the ancestral structural and functional domain [17]. However, they also show that the catalytic RNA-protein complex is ancient (Figure 5). The RPP contacts the catalytic domain of the RPR molecule, with specific contacts involving the P1-P4 core structure in a region that has been proposed to contain the active site and the phylogenetically conserved RNA core [43,59,71,82]. Evolutionary heat maps support the ancestrality of these contacts, which are basal on the trees of substructures (Figure 5B and 4C). The implication of a direct interaction of the RPP with the ancient P1-P4 core structure is therefore fundamental, suggesting the early involvement of proteins in catalysis.

It is noteworthy that both archaeal and eukaryal RPPs show extensive protein-protein and protein-RNA interactions, and that some of these may also involve the S domain. Furthermore, only a subset of the protein subunits may be necessary for catalytic activity while other proteins function in assembly and/or localization. In fungi, only two (Pop1p and Pop4p) out of nine nuclear RPPs interact with RPR [87]. Tsai et al. [88] suggested that the Rpp21/Rpp29 and Pop5/Rpp30 pairs in *Pyrococcus furiosus *interact with the S and C domains, respectively, enabling the inter-domain cooperation required for optimal pre-tRNA recognition and catalysis. Enzymatic footprinting also demonstrated that the RPP21/RPP29 protein complex in *Pyrococcus furiosus *interacts only with the S domain of the RPR [89]. Moreover, many studies show that helix P3 of the RPR catalytic core, which is ancient in our study, is the binding site for RPPs and RNase MRP proteins. Crystal structure of the P3 in RNase MRP of *Saccharomyces cerevisiae *also suggests some likely functions of P3 in stabilizing the enzyme's structure and in interactions with pre-tRNA [90]. In human nuclear RNase P, Rpp21 binds to H1 RNA and also to the P3 domain [91]. In *S. cerevisiae *RNase MRP and RNase P, Pop6 and Pop7 form a heterodimer that binds directly to P3, protecting a segment of the lower strand of the internal loop of P3 and part of the adjacent helical stem [92]. Similar results were obtained for human RNase MRP [93]. Furthermore, RNA-protein interactions involving P3 are not limited to the Pop6/Pop7 heterodimer; other proteins probably interact with this extended helix [94]. For example, helix P3 also appears to interact specifically with Pop1 in both *S. cerevisiae *RNase P and RNase MRP [95].

### The age and evolution of RPPs

The number of proteins associated with catalytic RNA varies greatly between Archaea, Eukarya, and Bacteria. In general, there are 4-5 and 9-10 protein units in archaeal [96] and eukaryal molecules [4,97-99], respectively. In contrast, bacterial RNase P enzymes are the simplest versions of the complex. They consist of only one protein and a single RPR molecule, providing a conserved and straightforward molecular ensemble for crystallographic study [60].

**Figure 6:**
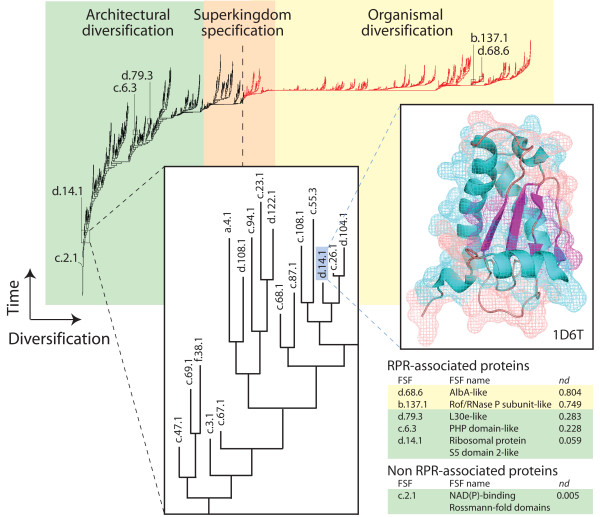
**Phylogenomic analysis of domain structure and the age of RPPs**. A universal tree of protein architecture was reconstructed from a genomic census of protein domain structure at FSF level of structural classification and RPPs of known structure were traced in the tree. The three evolutionary epochs of the protein world are colored with different shades and are overlapped to the trees, following definitions described in Wang et al. [37]. Terminal leaves are not labeled in the tree since they would not be legible. Branches in red delimit the birth of architectures after the appearance of the first architecture unique to a superkingdom (dashed line). Leaves corresponding to RPP FSFs are indicated and labeled using SCOP nomenclature. The inset shows a representative structure of the FSF architecture d.14.1.

In order to study the evolution of RPPs and determine the putative age of protein-RNA contacts, we timed the appearance of the 3D structure of RPP-associated domains in a tree of architectures derived from phylogenomic analysis of domain structure at FSF level of structural classification (Figure 6). The global phylogeny of protein architectures was reconstructed from a HMM-based genomic census in 584 completely sequenced organisms (Figure 6). This tree describes the history of 1,453 FSFs and was used to determine the relative age of RPP domains of known structure. We also used HMMs to assign protein structure to 1,136 RPP-associated sequences in the UniProtKB database, revealing that 1,029 of these entries were linked to 6 FSFs, 5 of which were RPR-associated holoenzymes. Interestingly, 5 sequence entries were linked to the NAD(P)-binding Rossmann fold domain (c.2.1), the second most ancient FSF (*nd *= 0.005), and corresponded to the recently identified RPPs that do not associate with RNA cofactors [100]. The age of RPR-linked domains ranged from *nd *= 0.06 in the ribosomal protein S5 domain-like domain (d.14.1) typically found in bacterial complexes to *nd *= 0.803 in the AlbA-like domain (d.68.6).

We previously identified three epochs in the evolution of proteins [37] and RNA [30], an ancient 'architectural diversification epoch' in which ancient molecules (including tRNA and 5S rRNA) [31,41] emerged and diversified, a 'superkingdom specification' epoch in which molecules sorted in emerging archaeal and eukaryal-like organismal lineages, and a late 'organismal diversification' epoch in which molecular lineages diversified in an increasingly diversified tripartite world. Four of all RPP domains (c.2.1, d.14.1, c.6.3, and d.79.3) originated in the architectural diversification epoch, while two appeared quite late during the organismal diversification epoch (c.6.3 and d.68.6) (Figure 6). Interestingly, the most ancient RPR-associated protein domain (*nd *= 0.06), the ribosomal protein S5 domain-like FSF (d.14.1) depicted in the complex of Figure 5B and 5C, appeared very early in the protein world, at the start of the architectural diversification epoch. Other RPR domains associated with known crystal structures directly through PDB entries or when using HMMs of structural recognition, were more derived (Figure 6), some even appearing during the organismal diversification epoch. This suggests RPPs interacted with the ancient P1-P4 core structure to form a primordial RNase P complex very early in evolution, at a time when the world of organisms was not diversified. Since the most ancient domain architectures in this tree had an origin in nucleotide metabolism [38,101], it is clear that this primordial RNase P complex was derived compared to ancient protein enzymes in primitive metabolic networks.

One interesting observation is the age of the domain linked to RNase P enzymes in organelles of Eukarya that do not require RPR cofactors (e.g., human mitochondrial RNase P) [100]. The domain of these protein-only RNAse P molecules, the NAD(P)-binding Rossmann fold domain (c.2.1) is widely distributed in nature (e.g. present throughout metabolism) and is very old (*nd *= 0.005). This suggests that the addition of the RPR moiety to the catalytic protein-based RNase P enzymes is either a derived feature or alternatively that the domain was co-opted late in evolution in eukaryotic organelles to perform the ribonucleoprotein task. However, the fact that the make-up of RPP domains is varied and evolutionarily diverse in RNase P suggests recruitment plays an important role in evolution of catalysis in this complex and the argument can be used to disfavor the idea that organellar protein enzymes are ancient fossils. Without additional evidence capable of dissecting recruitment, however, the age of the c.2.1 domain cannot be used to support or refute the ancestrality of RPPs relative to RPRs.

### The ancient origin and centrality of stem P12

Studies have shown that the S domain contributes to pre-tRNA recognition and helps position the substrate for optimal cleavage [10,102,103]. However, while RPPs appear to bind solely to the C domain [71], footprinting [104] and crosslinking analyses [105] suggest that the P12 substructure is also part of the protein-binding site. However, the possible role of P12 has been questioned; further studies have shown that the protein footprinting is restricted to the C domain [69,106,107]. It is noteworthy that the sequence at the base of helix P12 is relatively conserved and is physically adjacent to the core of the enzyme in close proximity to the connection between the top and bottom halves of the pre-tRNA molecules (see heat maps in Figure 5). However, the overall sequence of P12 does not contain any of the universally conserved nucleotides of the molecule [3,15] and its role as functional determinant is variable, being sometimes dispensable (e.g. in cyanobacteria [108]). Regardless of it playing an accessory or vital role in the current function of the molecule, the observation that P12 is indeed the most ancestral substructure of the complex (Figures 4 and 5), despite it being considered part of the structural S domain, lends support to its early role in a putative catalytic activity that is now partially displaced to the more derived P1-P4 core structure and also involves crucial protein-RNA interactions. This ancient catalytic activity could have been different to that of the extant catalytic RPR core and could have been neofunctionalized later on in evolution as the structure was co-opted to perform new roles. It is interesting to note that P12 has been lost in a few lineages, most probably as a secondary evolutionary event. This may suggest that its functional role is limited and sometimes dispensable and that in some cases the substructure does not contribute significantly to organismal fitness. Alternatively, the loss of the substructure may have been advantageous, as it could have defined different substrate specificities.

Results also underscore the significance of a primordial stem-loop that originally harbored a multitude of primordial functions, most of which were lost or displaced as molecules evolved and gained specific roles. The concept of a hairpin being the starting component of tRNA [109] has been emphasized by the genomic tag hypothesis [73] and has been recently supported by phylogenetic studies of the structure of SINE RNA [33] and tRNA molecules [30]. Recent analyses of 5S rRNA [41] and major rRNA subunits (Harish and Caetano-Anollés, unpublished) are in line with this evidence. The proposal that the P12 substructure of the RNA subunit may be a modern derivative of the primitive multifunctional hairpin structure and that this substructure probably lost most primordial functions as the RNase P complex evolved is therefore of great significance and merits careful examination.

## Conclusions

**Figure 7:**
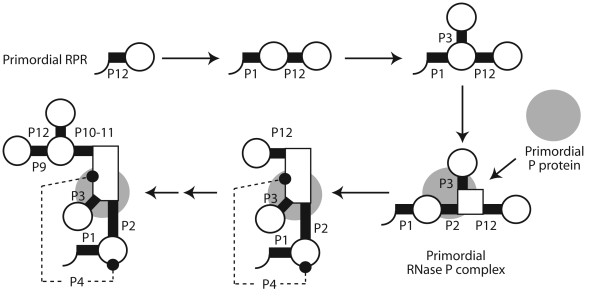
**A model of molecular evolution**. The model is derived directly from trees of RPR substructures and RPR-RPP contacts and shows formation of substructures homologous to present day helical regions in RPRs and interaction with primordial protein molecules during the course of evolution.

Our study reveals several important evolutionary patterns linked to the structure and function of RNA and protein components of RNase P: (i) the early origin of archaeal RPR molecules, which suggests the lineage leading to superkingdom Archaea is ancestral; (ii) the origin of the RPR molecule in the P12 substructure, closely followed by the catalytic P1-P4 core structure; (iii) the ancient origin of the C domain; (iv) the early appearance of RPP substructures that interact with proteins in this primordial RNase P complex; (v) and the ancestral nature of RPR-associated proteins, which originated at the onset of the protein world. Based on these observations we propose a model for the early evolution of the ribonuclease catalytic complex in a lineage leading to the last universal common ancestor of life (Figure 7). In this model, the interaction of primordial protein and RNA molecules result in a complex that is stabilized later in evolution by the establishment of a pseudoknotted structure (substructure P4). This catalytic structure is ultimately responsible for the modern make-up of the molecule, as accessory substructures organize around it and enhance the catalytic activities and specificity of the evolving ribonucleoprotein particles.

## Methods

### Data

The secondary structures of 130 RPR sequences were retrieved from the RNase P Database (http://jwbrown.mbio.ncsu.edu/RNaseP/home.html; Release No. 12, 2005 edition, [110]). Partial sequences were excluded in this study. Selected RNA sequences folded into secondary structures that are compatible with RPR phylogeny and known 3-dimensional models of RPR structure [58]. Another three RPRs from *Entamoeba histolytica *[111], *Zea mays *[45], and *Candidatus Kuenenia stuttgartiensis *[112] were also included to increase sampling diversity. Phylogenetic trees showed that one mitochondrial (*Reclinomonas americana*), two chloroplast (*Porphyra purpurea *and *Zea mays*), and one cyanelle (*Cyanophora paradoxa*) sequences were closely related to bacterial sequences, a result that is consistent with the evolutionary origins of these organelles. These four taxa were subsequently included in Bacteria. Table 1 shows the taxonomic distributions of the sampled RPRs. Note that RPR sequences sampled in this study are the only available that are associated to structure [42] and that their number is limited when compared to sequences available from metagenomic projects [113]. However, the set of RPR molecules selected encompasses all major RPR substructures and all major thematic variations that exist in molecules (Table 3). Consequently, our 133 RPR dataset does not exclude major structural designs and results should be impervious to sampling. The set also encompasses a relatively balanced selection of organisms representing the three superkingdoms. Because our study does not represent a systematic analysis to discriminate species, representative sampling is an appropriate strategy. We believe the species discrimination will likely improve by inclusion of more species, while the conclusions drawn will not change significantly. In fact, a ~50% reduction in the number of RPRs (from 133 to 69, with a RPR set that includes 39 bacterial, 15 archaeal and 15 eukaryal molecules) did not affect the conclusions of the analysis that we here present suggesting molecular sampling is appropriate.

### Phylogenetic characters

Overall, we scored a total of 129 structural characters in the 133 RPR molecules analyzed by comparative sequence analysis, comparison with crystallographic models, and other criteria (Additional file 1: Table S1). Character homology was determined by the relative position of substructures in the secondary structures (see below) and coded character states were based on the length (number of bases or base pairs) and number of these substructures. These characters (also referred to as 'geometrical' characters) describe the geometry of the molecules by measuring, for example, the length in nucleotides of each spatial component of secondary structure. These components include double helical stems, hairpin loops, bulges and interior loops, and unpaired segments such as 5' or 3' free ends, connecting joints, and multi-loop sequences separating stems. Character states were defined in alphanumerical format with numbers from 0 to 9, letters from A to Z and a to z, and other symbols (Table 2). Missing substructures were given the minimum state (0). The data matrix of RPR structure is given in Additional file 1: Table S2. Partitioned data matrices were built based on folding domains (S and C domains), types of characters (stabilizing characters such as stems, or de-stabilizing characters such as bulges, hairpins, and other single-stranded regions), or superkingdoms (Archaea, Bacteria, and Eukarya). Sequences were aligned based on the secondary structure models [42]. Eukarya and combined datasets were aligned using Clustal X [114] and adjusted manually. Statistics related to the sequences that were analyzed are described in Table 1.

### Character homology

The criterion of primary homology is based on the feature of structure being studied and its associated evolutionary model, and how this feature relates to the substructural taxa analyzed. Features can be descriptions of the geometry (e.g., shape characters) or the branching, stability, and plasticity (e.g., statistical characters) of homologous substructural components. In this study we focus on the former. Homologous substructures represent those that are of the same kind (e.g., domains, stems, base pairs) and respond to the same evolutionary model defining the character transformation sequences. For example, we reconstruct trees of coaxial stems corresponding to the helical regions in RPR, separate from trees of hairpin loops. This is because character change leading to coaxial stem taxa depends on models of character state that are quite different from those governing unpaired segments.

### Character argumentation

Structural features were treated as linearly ordered multistate characters that were polarized by invoking an evolutionary tendency towards molecular order. Establishing a preferred directionality of character state change resulted in intrinsically rooted trees, which were then used to define lineages that are either ancient or derived. Operationally, polarization was determined by fixing the direction of character state change using a transformation sequence that distinguishes ancestral states as those thermodynamically more stable. Maximum character states were defined as the ancestral states for stems and G:U base pairs (i.e. structures stabilizing the RPRs). Minimum states (0) were treated as the ancestral states for bulges, hairpin loops, and other unpaired regions (i.e. structures de-stabilizing the RPRs). The validity of character argumentation has been discussed in detail elsewhere [27,29-32,34,41,115], but is supported by a considerable body of theoretical and experimental evidence:

#### (i) Evidence from molecular mechanics

Many studies that focus on molecular mechanics strongly support a tendency towards molecular order. Comparative studies of extant and randomized sequences show that evolution enhances conformational order and diminishes conflicting molecular interactions [34,116-122]. Indeed, randomizations of mono- and di-nucleotides have been used to dissect the effects of composition and order of nucleotides in the stability of folded nucleic acid molecules and uncover evolutionary processes acting at RNA and DNA levels [123]. In recent bench experiments, extant evolved RNA molecules encoding complex and functional structural folds were compared to oligonucleotides corresponding to randomized counterparts [124]. Results show that arbitrary sequences, unlike evolved molecules, were prone to having multiple competing conformations. In contrast to arbitrary proteins, which rarely fold into well-ordered structures [125], these arbitrary RNA sequences were however quite soluble and compact and appeared delimited by physicochemical constraints such as nucleotide composition that were inferred in previous computational studies [120].

#### (ii) Evidence from thermodynamics

A molecular tendency towards order can be linked to fundamental concepts in thermodynamics [126]. The "building order from disorder" concept championed by Schrödinger [127] and others use energy dissipative processes linked to entropy to explain how energy that is able to do work (free energy) transforms into energy that is unavailable for that purpose. These processes fulfill the maximum entropy production principle (MEPP) advanced by Ziegler in non-equilibrium systems [128]. In this context, biological structure and organization acts as an engine that extracts, concentrates and stores free energy while maximizing the dissipation of energy gradients [129]. This optimization results in more efficient degradation of incoming (solar) energy through autocatalytic, self-assembly, reproduction, evolution and adaptation processes acting on molecular structures, all of which enhance the order of the system and are in line with second law of thermodynamics [130,131]. The optimization has also important consequences for evolution of molecular structure and the mapping of sequence to structure spaces, representing different levels of biological organization. For example, RNA molecules have low informational entropy in sequence space, but in structure space highly evolvable phenotypes are also more entropic [132]. These results suggest that increases in the order at one level of organization are counteracted by decreases in the order of the next. This relationship ultimately encourages escape (evolvability) from constraints of order (stasis through structural canalization). Note that a large body of theoretical evidence supports these sequence-to-structure mappings and their consequences on the energetic and kinetic landscape of the evolving molecules [133,134]. Furthermore, some important predictions have already been confirmed experimentally in *in vitro *evolution of ribozymes [135].

#### (iii) Evidence from cosmology

A tendency towards order is also supported by dissipation tendencies in energy and matter that exist in an open cosmological model of the Friedmann type [126]. This model describes that the universe expands faster than its contents can equilibrate, turning the nearly homogeneous hot gas at the beginning of the big bang into clumps of energy-dissipating matter that acquire more and more elaborate and finer-grained properties [136,137]. This emerging structure ultimately materializes in ordered structures and life. Note that three observational pillars support the big bang model: (1) the motion of galaxies away from each other, (2) the cosmic microwave background radiation, and (3) the relative quantities of light chemical elements (e.g. He, H) in cosmic gas.

#### (iv) Phylogenetic evidence

Finally and more importantly, tendencies towards structural order are experimentally supported by phylogenetic congruence of phylogenies reconstructed using geometrical and statistical structural characters [30,33,34] and of phylogenies derived from sequence, structure, and genomic rearrangements at different taxonomical levels [27,29,33,34,45,50,51]. These phylogenetic reconstructions are in line with traditional organismal classification. Remarkably, tests in which characters were polarized in the opposite direction generated phylogenetic trees that were less parsimonious and had topologies incompatible with accepted taxonomical knowledge [27,34]. Other more indirect results derived from using our focus on structure also proved to be congruent, such as hypotheses of organismal origin that used global trees of tRNA structures and constraint analysis [31] and phylogenies of proteomes derived from an analysis of protein structure in entire genomic complements [37]. Many new instances of congruence from ongoing phylogenetic studies (unpublished data) consistently support our hypothesis of polarization. Note that order is seldom achieved in frustrated molecular systems that are driven by the energetics of conformation and stability, and that while the proposed generalized trend in structure appears valid by the evidence outlined above, we do not know the nature and stability of selective preferences or constraints acting on primordial RNA during the early stages of evolution of these molecules.

### Phylogenetic analysis

Data matrices were analyzed using equally weighted MP as the optimality criterion in PAUP* [138]. Note that a more realistic weighting scheme should consider for example the evolutionary rates of change in structural features. However, this requires the measurement of evolutionary parameters along individual branches of the tree and the development of an appropriate quantitative model. In the absence of this information, it is most parsimonious and preferable to give equal weight to the relative contribution of each character. The use of MP (the preference of solutions that require the least amount of change) is particularly appropriate and can outperform maximum likelihood (ML) approaches in certain circumstances [139]. MP is precisely ML when character changes occur with equal probability but rates vary freely between characters in each branch. This model is useful when there is limited knowledge about underlying mechanisms linking characters to each other [139]. Furthermore, the use of large multi-step character state spaces decreases the likelihood of revisiting a same character state on the underlying tree, making MP statistically consistent. Phylogenetic analyses of stem characters common to all three superkingdoms (either P1 to P4, P10, and P11, or P1 to P4, P7, and P9-P11) were conducted to investigate the phylogenetic utility of the conserved helical components of RPR. These stem structures are a major subset of the core structures defined by Siegel et al. [19]. Depending on the number of taxa in each matrix, tree reconstructions were sought using either exhaustive, branch-and-bound, or heuristic search strategies. When the heuristic search strategy was used, 1,000 heuristic searches were initiated using random addition starting taxa, with tree bisection reconnection (TBR) branch swapping and the MULTREES option selected. One shortest tree was saved from each search. Hypothetical ancestors were included in the searches for the MP trees using the ANCSTATES command. A "total evidence" approach [140,141], also called "simultaneous analysis" [142], was applied in phylogenetic analyses to combine both sequence and structure data of the complete and partitioned matrices. The goal of this analysis was to provide stronger support for the phylogenetic groupings recovered from analyses of structural data. For comparison, a distance-based phylogenetic method (i.e., neighboring-joining) was also performed on all matrices. Bootstrap support (BS) values [143] were calculated from 10^5 ^replicate analyses using "fast" stepwise addition of taxa in PAUP*. The g_1 _statistic of skewed tree length distribution calculated from 10^4 ^random parsimony trees was used to assess the amount of nonrandom structure in the data [144].

Evolutionary relationships derived from trees of substructures were traced in generic 2-dimensional models of RPR secondary structure that we here call *evolutionary heat maps of ancestry*. Because reconstructed trees were intrinsically rooted, we established the relative age (ancestry) of each substructure by measuring a distance in nodes from the hypothetical ancestor on a relative 0-1 scale. To do this, we counted the number of nodes in every lineage from the root to the terminals of the tree and divided this number by the maximum number of nodes in a lineage [28]. Ancestry values were divided in classes, giving them individual hues in a color scale that was then used to color substructures in the proposed RPR secondary structure model.

### Phylogenomic analysis of protein architecture

A census of the genomic sequence of 584 organisms, including 46 Archaea, 397 Bacteria and 141 Eukarya, assigned protein structural domains corresponding to 1,453 fold superfamilies (FSFs) to protein sequences using advanced linear hidden Markov models (HMMs) of structural recognition in SUPERFAMILY and a probability cutoff *E *of 10^-4^. FSFs were defined by the SCOPhttp://scop.mrc-lmb.cam.ac.uk/scop/ version 1.69 [145]. The census was used to build data matrices of genomic abundance of FSFs, which were coded as linearly ordered multistate phylogenetic characters. Data matrices were used to build universal trees of protein architectures with established methodology [146]. The reconstruction of these large trees is computationally hard and their visualization challenging. We used a combined parsimony ratchet (PR) and iterative search approach to facilitate tree reconstruction [36]. A recent review summarizes the general approach and the progression of census data and tree reconstruction in recent years [98]. In order to discover architectures associated with RPPs, we queried the UniProtKB (Protein Knowledgebase) database http://www.uniprot.org/ and downloaded 1,136 protein sequences in Fasta format. HMMs were then used to predict the SCOP identifiers (IDs) describing individual FSFs linked to the sequences. We finally used the "SCOP parseable files" link in SCOP to identify the corresponding IDs in the "dir.des.scop.txt 1.69" file. PROTEIN DATA BANK (PDB) files associated with RPPs were queried and downloaded from the PDB database http://www.rcsb.org/pdb/home/.

## Authors' contributions

Both authors conceived, designed, and performed experiments, analyzed the data, and wrote the article.

## Supplementary Material

Additional file 1 Figure S1 Phylogenetic trees of RPR molecules derived from the C domain. Figure S2 Phylogenetic trees of RPR molecules derived from the S domain. Figure S3 Phylogenetic trees of RPR molecules derived from a conserved substructural core. Figure S4 Phylogenetic trees of RPR molecules derived from a conserved substructural core. Figure S5 Phylogenetic trees of stem substructures derived from the C and S domains of the RPR molecule. Figure S6 Cumulative frequency distribution plot of molecular substructures. Table S1 Structural characters and their statistics (range and mean ± standard deviation) used in phylogenetic analyses. Table S2 Data matrix of structural characters used in the cladistic analyses for RPR molecules. Click here for file
