# Supplementary material for: The ancient history of the structure of ribonuclease P and the early origins of Archaea
Source: BMC Bioinformatics. 2010 Mar 24;11:153. doi: 10.1186/1471-2105-11-153 (PMC2858038; doi:10.1186/1471-2105-11-153)
Supplement: Additional file 1 — Figure S1 Phylogenetic trees of RPR molecules derived from the C domain. Figure S2 Phylogenetic trees of RPR molecules derived from the S domain. Figure S3 Phylogenetic trees of RPR molecules derived from a conserved substructural core. Figure S4 Phylogenetic trees of RPR molecules derived from a conserved substructural core. Figure S5 Phylogenetic trees of stem substructures derived from the C and S domains of the RPR molecule. Figure S6 Cumulative frequency distribution plot of molecular substructures. Table S1 Structural characters and their statistics (range and mean ± standard deviation) used in phylogenetic analyses. Table S2 Data matrix of structural characters used in the cladistic analyses for RPR molecules. [file 1471-2105-11-153-S1.PDF]

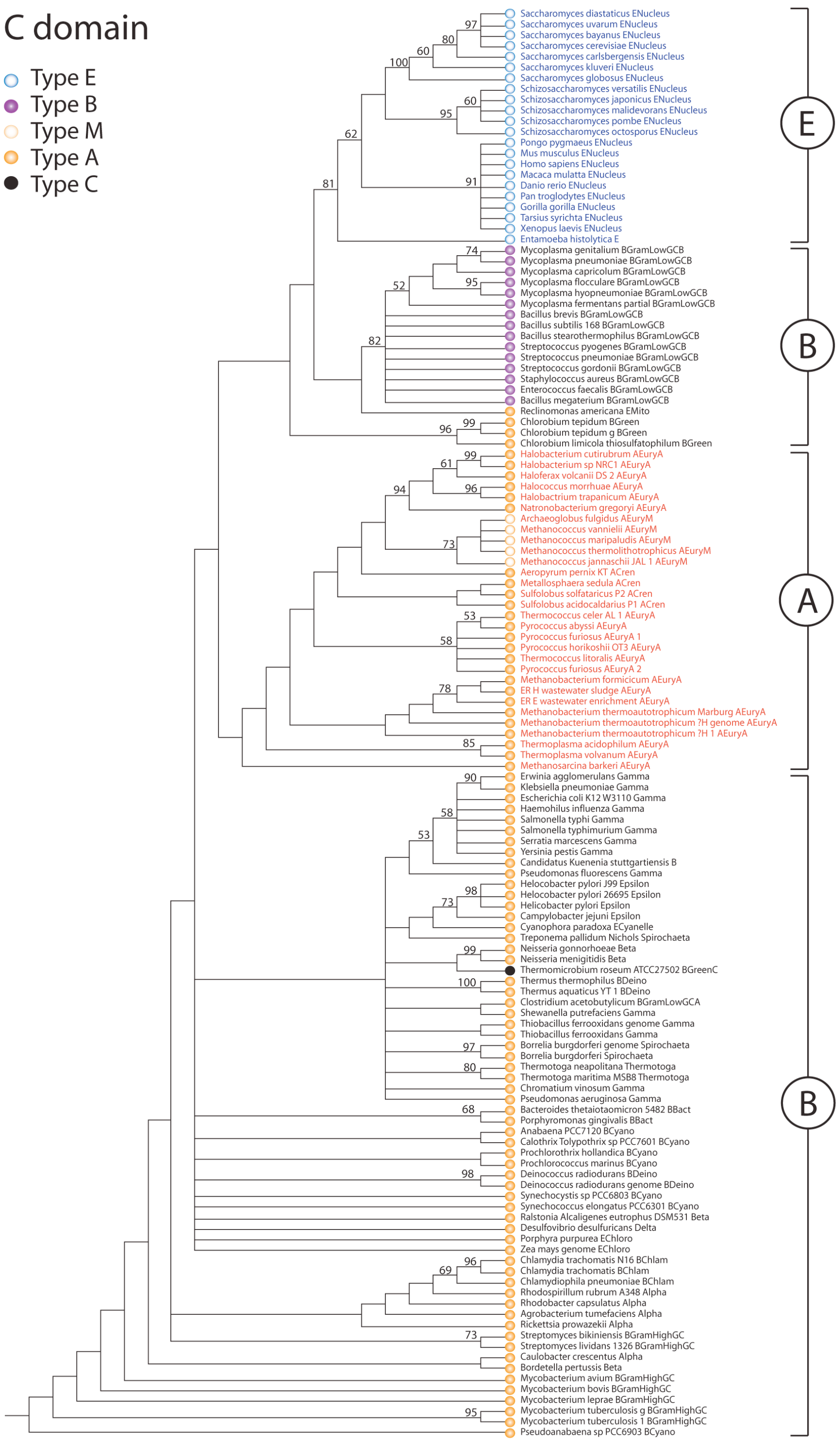

Figure S1. Phylogenetic trees of RPR molecules derived from the C domain. MP analyses of 87 structural characters describing geometrical features of the C domain resulted in the preset limit of 10,000 minimal length trees, each of 2,342 steps (CI = 0.253 or 0.249, with or without informative characters; RI = 0.830; RC = 0.210; $g_1 = -0.238$). BS >50% are shown for individual nodes. Symbols in terminal leaves describe the structural type of the RPR molecules. A, Archaea; B, Bacteria; E, Eukarya.

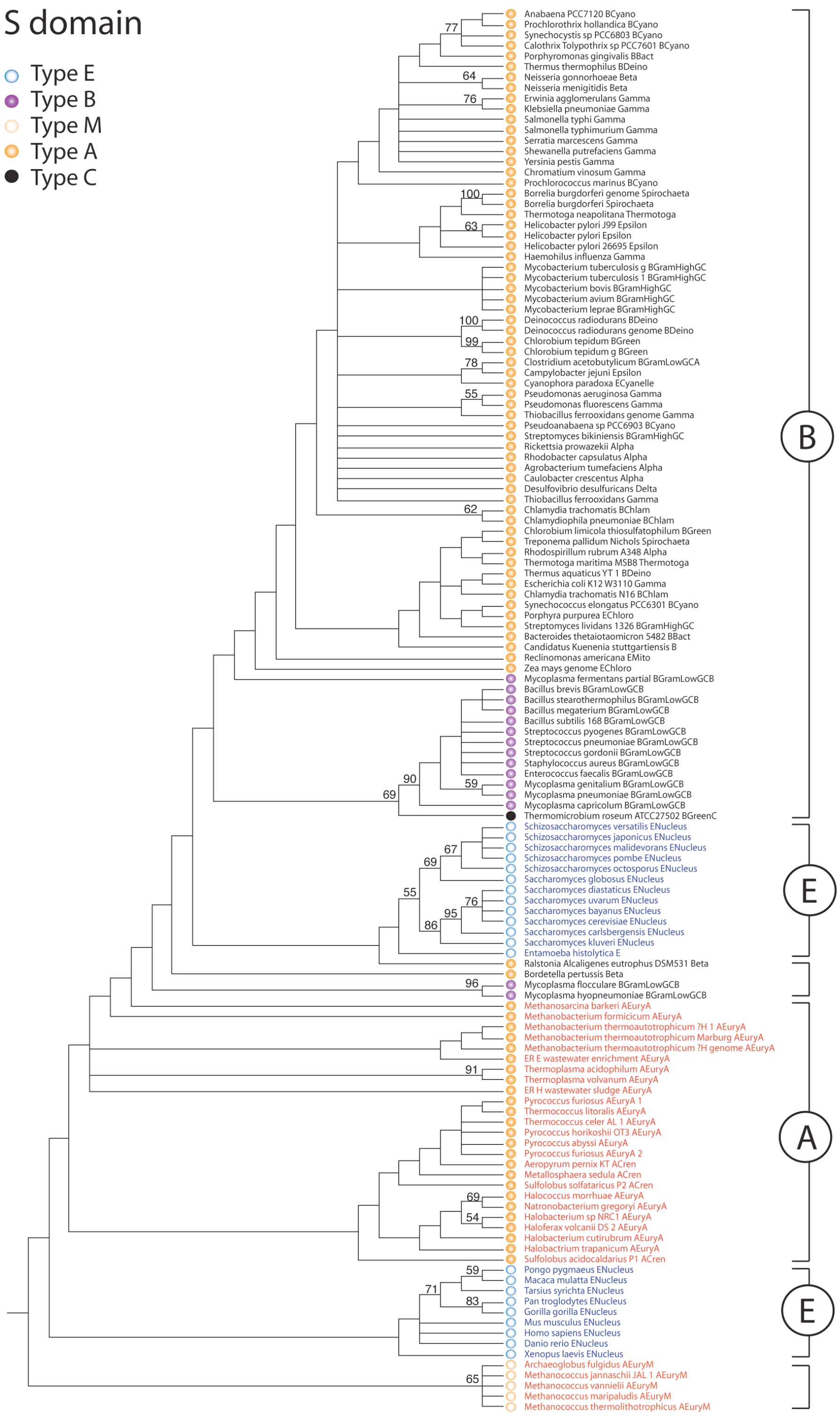

Figure S2. Phylogenetic trees of RPR molecules derived from the S domain. MP analyses of 42 structural characters describing geometrical features of the C domain resulted in the preset limit of 10,000 minimal length trees, each of 1,478 steps (CI = 0.316; RI = 0.845; RC = 0.267; g_1 = –0.212). BS >50% are shown for individual nodes. Symbols in terminal leaves describe the structural type of the RPR molecules. A, Archaea; B, Bacteria; E, Eukarya.

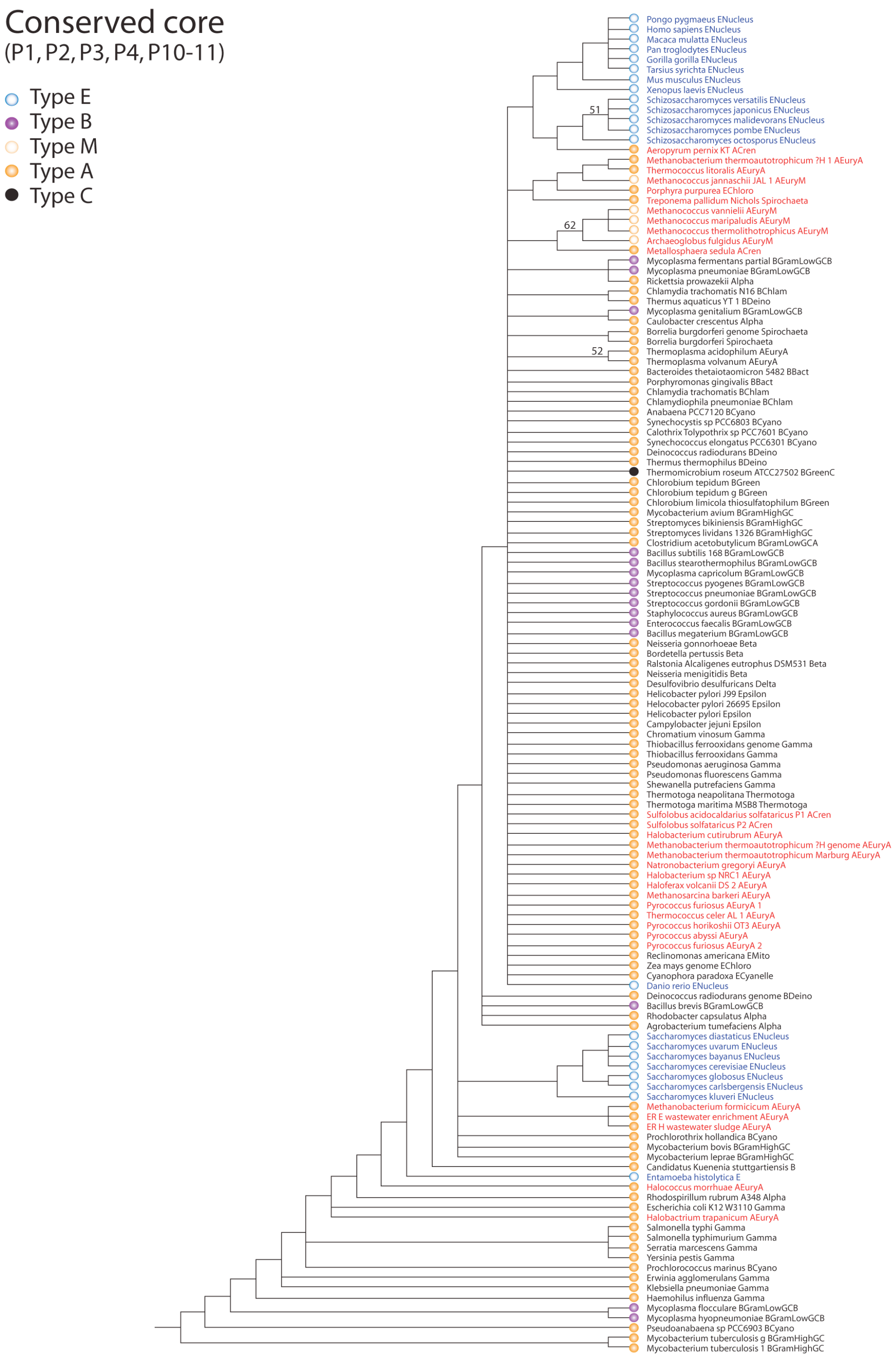

Figure S3. Phylogenetic trees of RPR molecules derived from a conserved substructural core. Strict consensus of 5,000 most parsimonious trees of molecules (193 steps; CI = 0.264, RI = 0.852; RC = 0.225; g_1 = –0.142) reconstructed from a conserved substructural core that included the P1, P2, P3, P4, and P10-11 substructures. BS >50% are shown for individual nodes. Symbols in terminal leaves describe the structural type of the RPR molecules.

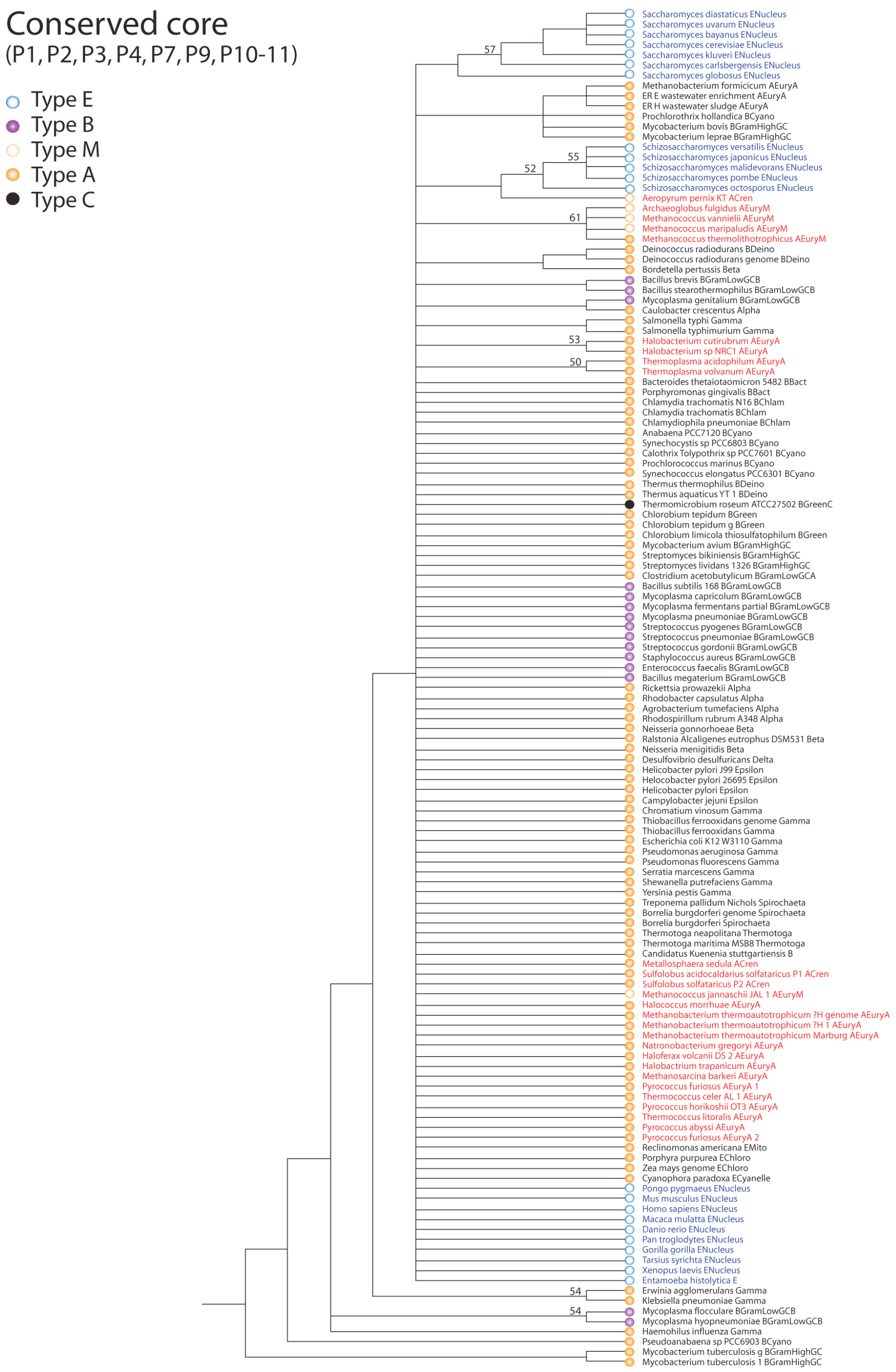

Figure S4. Phylogenetic trees of RPR molecules derived from a conserved substructural core. Strict consensus of 5,000 most parsimonious trees of molecules (281 steps; CI = 0.246, RI = 0.811; RC = 0.199; g_1 = –0.139) reconstructed from a conserved substructural core that included the P1, P2, P3, P4, P7, P9, and P10-11 substructures. BS >50% are shown for individual nodes. Symbols in terminal leaves describe the structural type of the RPR molecules.

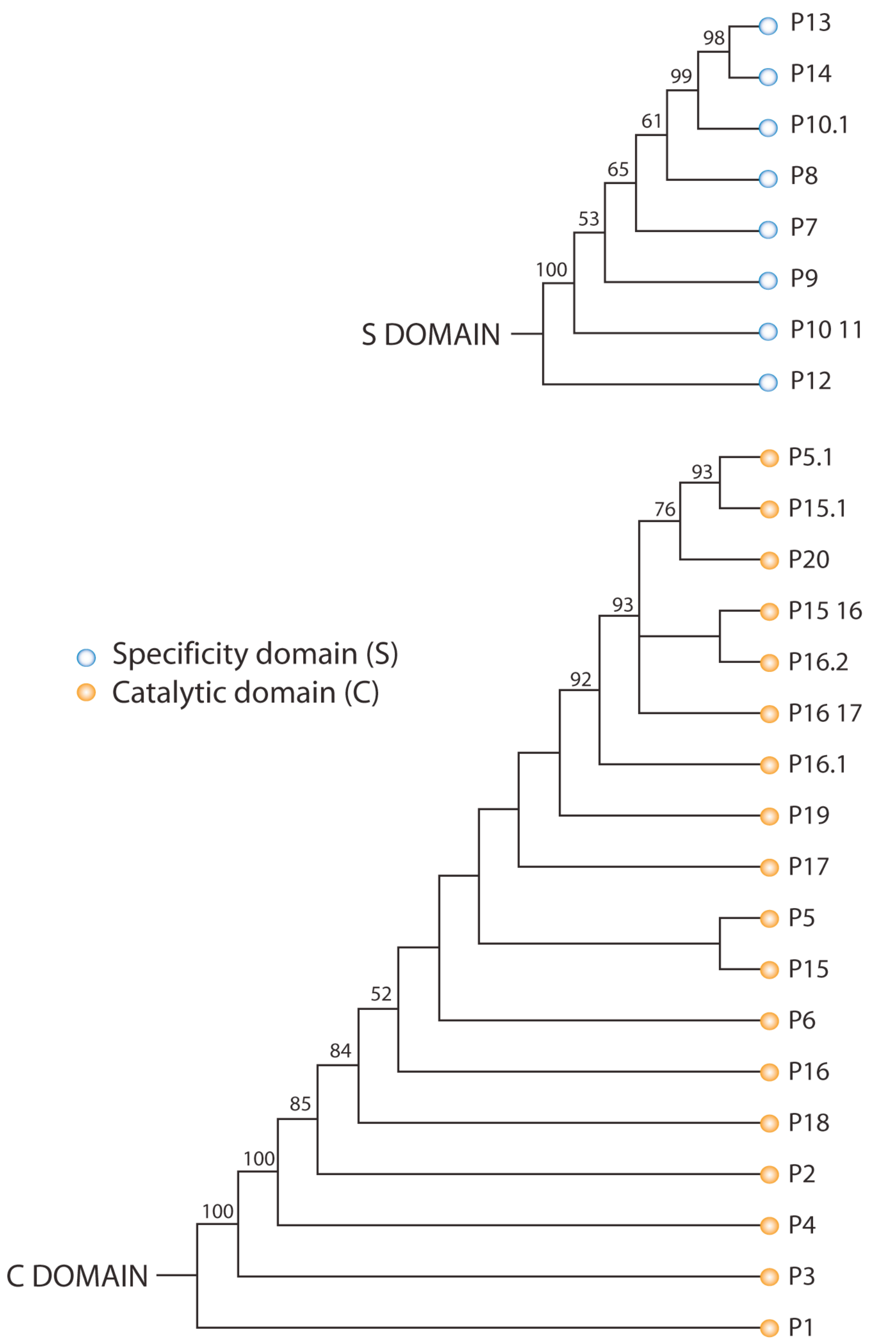

Figure S5. Phylogenetic trees of stem substructures derived from the C and S domains of the RPR molecule. MP analyses recovered one minimal tree (10,118 steps; CI = 0.946, RI = 0.799; RC = 0.756; g_1 = –2.435) or two trees of substructures (11,293 steps; CI = 0.848, RI = 0.767; RC = 0.651; g_1 = –0.653) reconstructed from characters describing geometrical features of the S and C structural domains of the RPR molecule, respectively. BS >50% are shown for individual nodes. Symbols in terminal leaves describe substructures in structural domains.

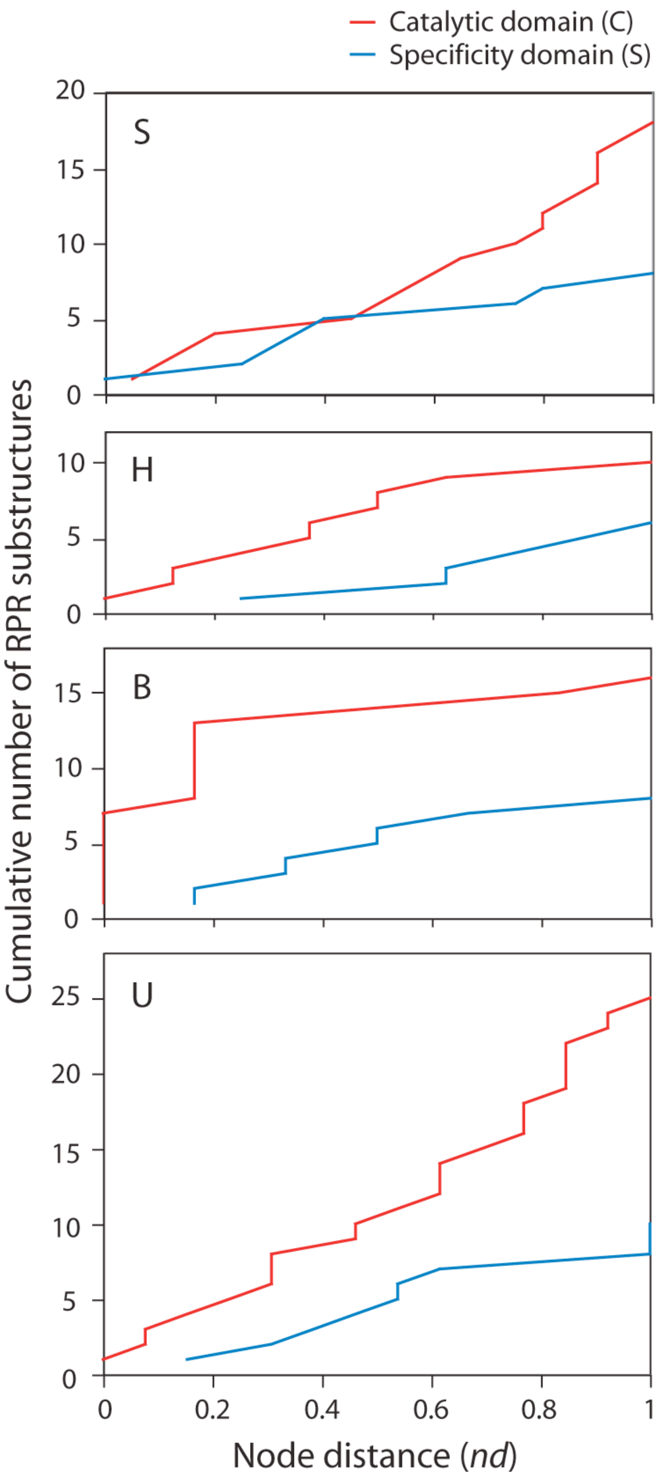

Figure S6. Cumulative frequency distribution plot of molecular substructures. The plot describes the accumulation of stems (S), hairpin loops (H), bulges and internal loops (B), and unpaired segments (U) specific to the C and S domains along a timeline defined by the distance (*nd*) in nodes from the ancestral substructure at the base of the tree, on a relative scale. Data were obtained from trees described in Figure 3.

Table S1. Structural characters and their statistics (range and mean ± standard deviation) used in phylogenetic analyses. Characters were scored along the 5'- to 3'-end direction of the molecules and their names are abbreviated for use in phylogenetic trees. Character states of these polymorphic characters are indicated as numbers 0-9, letters A-W, and symbol "+".

Characters	Character abbreviations	Character states
1. Number of unpaired bases of 5' free end on P1	U5end	0 – 6 (0.4 ± 1.0)
2. Number of unpaired bases of 3' free end on P1	U3end	0 – 9 (2.7 ± 2.2)
3. Length of P1 (number of bases)	P1	5 – L (11.8 ± 2.9)
4. Length of bulges (number of bases) of 5' side on P1	B 4	0 – 3 (0.5 ± 0.7)
5. Length of bulges (number of bases) of 3' side on P1	B 5	0 – 8 (0.9 ± 1.0)
6. Number of unpaired bases between P1 and P2	U1 2	0 – 3 (0.5 ± 0.9)
7. Length of P2 (number of bases)	P2	4 – 7 (6.4 ± 0.8)
8. Length of bulges (number of bases) of 5' side on P2	B 8	0 – 1 (0.0 ± 0.2)
9. Length of bulges (number of bases) of 3' side on P2	B 9	0 – 1 (0.0 ± 0.2)
10. Number of unpaired bases between P2 and P3	U2 3	0 – 9 (2.1 ± 1.9)
11. Length of P3 (number of bases)	P3	2 – O (10.1 ± 6.5)
12. Length of bulges (number of bases) of 5' side on P3	B 12	0 – B (2.2 ± 3.6)
13. Length of bulges (number of bases) of 3' side on P3	B 13	0 – D (2.6 ± 4.2)
14. Length of hairpin loop (number of bases) of H3	H3	3 – A (4.7 ± 1.6)
15. Number of unpaired bases between P3 and P4	U3 4	1 – 8 (4.5 ± 1.2)
16. Length of P4 (number of bases)	P4	5 – 9 (7.7 ± 0.9)
17. Length of bulges (number of bases) of 5' side on P4	B 17	0 – 1 (0.9 ± 0.4)
18. Length of bulges (number of bases) of 3' side on P4	B 18	0 (0.0 ± 0.0)
19. Number of unpaired bases between P4 and P5	U4 5	0 – 5 (0.4 ± 1.3)
20. Length of P5 (number of bases)	P5	0 – 7 (3.9 ± 1.2)
21. Length of bulges (number of bases) of 5' side on P5	B 21	0 – 1 (0.0 ± 0.1)
22. Length of bulges (number of bases) of 3' side on P5	B 22	0 – 1 (0.0 ± 0.1)
23. Number of unpaired bases between P5 and P5.1	U5 5.1	0 (0.0 ± 0.0)
24. Number of unpaired bases between P5 and P6	U5 6	0 – 6 (1.9 ± 1.5)
25. Length of P5.1 (number of bases)	P5.1	0 – 6 (0.7 ± 1.9)
26. Length of bulges (number of bases) of 5' side on P5.1	B 26	0 – 1 (0.0 ± 0.1)
27. Length of bulges (number of bases) of 3' side on P5.1	B 27	0 (0.0 ± 0.0)
28. Length of hairpin loop (number of bases) of H5.1	H5.1	0 – 6 (0.7 ± 1.9)
29. Length of P6 (number of bases)	P6	0 – 8 (3.5 ± 2.7)
30. Length of bulges (number of bases) of 5' side on P6	B 30	0 (0.0 ± 0.0)
31. Length of bulges (number of bases) of 3' side on P6	B 31	0 (0.0 ± 0.0)
32. Number of unpaired bases between P5.1 and P7	U5.1 7	0 – 5 (0.6 ± 1.6)
33. Number of unpaired bases between P6 and P7	U6 7	0 – 3 (0.8 ± 0.6)
34. Length of P7 (number of bases)	P7	3 – 7 (4.8 ± 0.6)
35. Length of bulges (number of bases) of 5' side on P7	B 35	0 – 1 (0.1 ± 0.4)
36. Length of bulges (number of bases) of 3' side on P7	B 36	0 – 1 (0.1 ± 0.2)
37. Number of unpaired bases between P7 and P8	U7 8	0 – Q (2.1 ± 5.2)
38. Length of P8 (number of bases)	P8	0 – C (5.1 ± 2.1)
39. Length of bulges (number of bases) of 5' side on P8	B 39	0 – 2 (0.0 ± 0.2)
40. Length of bulges (number of bases) of 3' side on P8	B 40	0 – 2 (0.1 ± 0.4)

41. Length of hairpin loop (number of bases) of H8	H8	0 – A (4.8 ± 2.2)
42. Number of unpaired bases between P8 and P9	U8 9	0 – F (0.7 ± 3.0)
43. Length of P9 (number of bases)	P9	2 – G (5.8 ± 2.0)
44. Length of bulges (number of bases) of 5' side on P9	B 44	0 – 7 (0.4 ± 1.5)
45. Length of bulges (number of bases) of 3' side on P9	B 45	0 – 8 (0.7 ± 1.1)
46. Length of hairpin loop (number of bases) of H9	H9	3 – B (4.3 ± 1.2)
47. Number of unpaired bases between P9 and P10-11	U9 10 11	0 − 8 (0.5 ± 1.8)
48. Number of unpaired bases between P10 and P10.1	U10 10.1	0 – A (0.2 ± 0.9)
49. Number of unpaired bases between P10.1 and P11	U10.1 11	0 – 5 (0.4 ± 1.3)
50. Length of P10-11 (number of bases)	P10 11	4 – A (5.3 ± 1.5)
51. Length of bulges (number of bases) of 5' side on P10-11	B 51	0 – 4 (2.1 ± 1.4)
52. Length of bulges (number of bases) of 3' side on P10-11	B 52	0 – 2 (0.4 ± 0.7)
53. Number of unpaired bases between P10 and P7	U10 7	0 – 4 (0.3 ± 0.9)
54. Length of P10.1 (number of bases)	P10.1	0 − C (1.0 ± 3.1)
55. Length of bulges (number of bases) of 5' side on P10.1	B 55	0 – A (0.7 ± 2.1)
56. Length of bulges (number of bases) of 3' side on P10.1	B 56	0 – 9 (0.6 ± 2.0)
57. Length of hairpin loop (number of bases) of H10.1	H10.1	0 – 5 (0.4 ± 1.3)
58. Number of unpaired bases between P11 and P12	U11 12	A – W (13.5 ± 2.2)
59. Length of P12 (number of bases)	P12	0 − + (15.1 ± 10.8)
60. Length of bulges (number of bases) of 5' side on P12	B 60	0 – M (4.1 ± 5.8)
61. Length of bulges (number of bases) of 3' side on P12	B 61	0 – O (4.4 ± 4.9)
62. Length of hairpin loop (number of bases) of H12	H12	0 – J (5.5 ± 3.3)
63. Degree of multiloop on P12	DLoop12	0 – 6 (0.4 ± 1.3)
64. Number of unpaired bases along the multiloop on P12	MLoop12	0 – Q (1.3 ± 4.4)
65. Number of unpaired bases between P12 and P13	U12 13	0 – E (8.0 ± 2.3)
66. Length of P13 (number of bases)	P13	0 – 9 (2.7 ± 3.0)
67. Length of bulges (number of bases) of 5' side on P13	B 67	0 – 1 (0.1 ± 0.2)
68. Length of bulges (number of bases) of 3' side on P13	B 68	0 − C (0.2 ± 1.1)
69. Length of hairpin loop (number of bases) of H13	H13	0 – 7 (3.2 ± 3.4)
70. Number of unpaired bases between P13 and P14	U13 14	0 – 3 (0.5 ± 0.8)
71. Length of P14 (number of bases)	P14	0 – B (3.7 ± 4.0)
72. Length of bulges (number of bases) of 5' side on P14	B 72	0 – 2 (0.1 ± 0.3)
73. Length of bulges (number of bases) of 3' side on P14	B 73	0 – 3 (0.3 ± 0.6)
74. Length of hairpin loop (number of bases) of H14	H14	0 – 6 (1.9 ± 2.0)
75. Number of unpaired bases between P14 and P11	U14 11	0 – A (3.5 ± 4.3)
76. Number of unpaired bases between P7 and P5	U7 5	0 – K (1.4 ± 4.9)
77. Number of unpaired bases between P5 and P15	U5 15	0 – G (3.3 ± 2.9)
78. Length of P15-16 (number of bases)	P15 16	0 – B (0.4 ± 1.9)
79. Length of P15 (number of bases)	P15	0 – C (3.4 ± 2.4)
80. Length of bulges (number of bases) of 5' side on P15	B 80	0 – 2 (0.1 ± 0.4)
81. Length of bulges (number of bases) of 3' side on P15	B 81	0 – 3 (0.2 ± 0.6)
82. Length of hairpin loop (number of bases) of H15	H15	0 – B (1.7 ± 3.6)
83. Number of unpaired bases between P15 and P16	U15 16	0 – 7 (3.3 ± 2.9)
84. Number of unpaired bases between P16 and P15	U16 15	0 – F (3.6 ± 3.3)
85. Length of P16-17 (number of bases)	P16 17	0 – 4 (0.1 ± 0.6)
86. Length of P16 (number of bases)	P16	0 – 9 (3.5 ± 3.0)
87. Length of bulges (number of bases) of 5' side on P16	B 87	0 (0.0 ± 0.0)
88. Length of bulges (number of bases) of 3' side on P16	B 88	0 (0.0 ± 0.0)
89. Length of P16.1 (number of bases)	P16.1	0 – J (1.5 ± 3.2)

90. Length of bulges (number of bases) of 5’ side on P16.1	B 90	0 − 3 (0.0 ± 0.3)
91. Length of bulges (number of bases) of 3’ side on P16.1	B 91	0 – 4 (0.1 ± 0.4)
92. Length of hairpin loop (number of bases) of H16.1	H16.1	0 – 6 (0.9 ± 1.8)
93. Length of P16.2 (number of bases)	P16.2	0 – A (0.1 ± 0.9)
94. Length of bulges (number of bases) of 5’ side on P16.2	B 94	0 – 1 (0.0 ± 0.1)
95. Length of bulges (number of bases) of 3’ side on P16.2	B 95	0 – 1 (0.0 ± 0.1)
96. Length of hairpin loop (number of bases) of H16.2	H16.2	0 – 4 (0.0 ± 0.4)
97. Number of unpaired bases between P16 and P17	U16 17	0 – A (1.8 ± 2.0)
98. Number of unpaired bases between P17 and P16	U17 16	0 – 7 (0.5 ± 1.4)
99. Number of unpaired bases between P17 and P16.1	U17 16.1	0 – 6 (0.3 ± 1.0)
100. Number of unpaired bases between P16.1 and P16	U16.1 16	0 – 8 (0.3 ± 1.1)
101. Length of P17 (number of bases)	P17	0 – 6 (2.4 ± 2.1)
102. Length of bulges (number of bases) of 5’ side on P17	B 102	0 – 1 (0.0 ± 0.2)
103. Length of bulges (number of bases) of 3’ side on P17	B 103	0 – 1 (0.0 ± 0.2)
104. Length of hairpin loop (number of bases) of H17	H17	0 – 8 (1.2 ± 1.4)
105. Number of unpaired bases between P15 and P15.1	U15 15.1	0 – D (0.7 ± 2.1)
106. Number of unpaired bases between P15 and P18	U15 18	0 – E (6.2 ± 4.6)
107. Length of P15.1 (number of bases)	P15.1	0 – A (0.9 ± 2.4)
108. Length of bulges (number of bases) of 5’ side on P15.1	B 108	0 (0.0 ± 0.0)
109. Length of bulges (number of bases) of 3’ side on P15.1	B 109	0 (0.0 ± 0.0)
110. Length of hairpin loop (number of bases) of H15.1	H15.1	0 – I (1.9 ± 5.2)
111. Length of P18 (number of bases)	P18	0 – F (4.5 ± 4.0)
112. Length of bulges (number of bases) of 5’ side on P18	B 112	0 – 1 (0.1 ± 0.3)
113. Length of bulges (number of bases) of 3’ side on P18	B 113	0 – 3 (0.2 ± 0.4)
114. Length of hairpin loop (number of bases) of H18	H18	0 – C (2.4 ± 2.2)
115. Number of unpaired bases between P18 and P2	U18 2	0 – A (5.5 ± 4.7)
116. Number of unpaired bases between P2 and P19	U2 19	0 – G (5.2 ± 5.1)
117. Length of P19 (number of bases)	P19	0 – N (5.1 ± 6.5)
118. Length of bulges (number of bases) of 5’ side on P19	B 118	0 – 3 (0.4 ± 1.5)
119. Length of bulges (number of bases) of 3’ side on P19	B 119	0 – 8 (0.6 ± 2.2)
120. Length of hairpin loop (number of bases) of H19	H19	0 – 8 (2.3 ± 2.3)
121. Degree of multiloop on P19	DLoop19	0 – 3 (0.0 ± 0.3)
122. Number of unpaired bases along the multiloop on P19	MLoop19	0 – 3 (0.0 ± 0.3)
123. Number of unpaired bases between P19 and P4	U19 4	0 – B (3.7 ± 3.6)
124. Number of unpaired bases between P4 and P1	U4 1	0 – 7 (1.9 ± 1.6)
125. Number of unpaired bases between P1 and P20	U1 20	0 – 8 (0.2 ± 1.0)
126. Length of P20 (number of bases)	P20	0 – E (0.5 ± 2.4)
127. Length of bulges (number of bases) of 5’ side on P20	B 127	0 – 1 (0.0 ± 0.1)
128. Length of bulges (number of bases) of 3’ side on P20	B 128	0 – 1 (0.0 ± 0.1)
129. Length of hairpin loop (number of bases) of H20	H20	0 − 8 (0.3 ± 1.1)

Table S2. Data matrix of structural characters used in the cladistic analyses for RPR molecules. Taxa are grouped according to superkingdom (number of molecules is given in parentheses). Detailed descriptions of these characters are given in Table 2.

Taxon	RPR structural characters (20 · 40 · 60 · 80 · 100 · 120)
**Bacteria (77)**	
Bacteroides thetaiotaomicron 5482	04B0107001611548100400030000400015000411506004000720000000BB02400760060812410304000760700000000005100400106000071114A07003006100000
Porphyromonas gingivalis	05A0107001800448100400030000400015000500506004000420000000EK74400760072800480304000760600000000006200400106000071114A0A014006100000
Chlamydia trachomatis 1	04C0107001C34348100400030000400015000500505014000732000000CC024008600709014103020001508006004000010344002060000800 4A0C124006100000
Chlamydia trachomatis 2	04C0007001B45348100400030000400015000500505014000430000000FC02400370051800480302000150800600400001034400206000080 04A0C124006100000
Chlamydiophila pneumoniae	06D0107001B11648100400030000400015000500505014000430000000FB0240087005171148030200015050040040000505840020600000 7114A0D014006100000
Anabaena PCC7120	14D3117001A11348100500010000700015100500505014000430000000EaAC30076007270049030300 04C0500000000000200030020600008004A0A014007300000
Synechocystis sp. PCC6803	07B1007001A01348100500010000700015100500505014000430000000EVCA3007600727004903030005E050000000000020002003060000 8004A08113007200000
Prochlorothrix hollandica	00G2327001D01348100411020000600015100500505014000430000000Ed3A300760072700490303000490500A11400002000300506000080 04A08224007200000
Calothrix (*Tolypothrix*) sp. PCC7601	26D3107001901748100500010000700015100500505014000430000000EW7A8007600727004903030005C00000000000020003002060000 8004A0G134007100000
Prochlorococcus marinus	00B1007001H12448100411020000600015100500505014000430000000EA23300760072700490303000380200000000000330040060400009 114808114007200000
Pseudoanabaena sp. PCC6903	01D0106111L22448100500020000700015000400705014000430000000E9024007600727004903030004709 00J22400001000300606000080 04A18104009100000
Synechococcus elongatus PCC6301 (*Anacystis nidulans*)	29A2206111A01448100500010000700015100500505014000732000000BC23400760070901410303000490500000000000200020020600008 004A07003007100000
Deinococcus radiodurans	04A0017001A21548100400030000400025000500 50F78B00043000000FC03G0076007280049030400076050000000000260040010600008104A28114008300000
Deinococcus radiodurans g	04A0017001D00548100400030000400025000500 50G78B00043000000FB14G0076007280049030400076050000000000260040010600008104A29006008300000
Thermus thermophilus	40C0107001A00648100400030000400015000500505014000430000000FF12400751171900490304000660500000000002700500106000080 04A90000000137004
Thermus aquaticus YT	20E0007001A00648100400030000400015000500505014000732000000CF12300760070A01420304000660500000000002700500106000080 04AA0000000218004
Thermomicrobium roseum ATCC27502	04B0107001700548100400030000400014100600506009 0A170208005D50050090000000000003040004507000000000005000400130900480058A0000000100000
Chlorobium tepidum	00B1107001900348100400030000400014100902 80A02400043000000F9026007511727114903040006507006004000022004001D000000000018005007100000
Chlorobium tepidum g	00B1107001900348100400030000400014100902 80A02400043000000F9026007511727114903040006507006004000022004001D000000000018005007100000
Chlorobium limicola thiosulfatophilum	04B110700190054810040003000040001410 0A2230A02400073200000C913400751170912410304000650600700400003100 4001D000000000007225006100000
Mycobacterium tuberculosis g	00F0107001J13648100400030000400015000500505014000430000000F80250076007280049030400065060080050000400060010600008 01491F103007100000
Mycobacterium tuberculosis	00F0107001J13648100400030000400015000500505014000430000000F80250076007280049030400065060080050000400060010600008 01491F103007100000
Mycobacterium bovis	00F0107001E22448100400030000400015000500505014000430000000F80250076007280049030400075060080050000400060010600008 01491F103007100000
Mycobacterium avium	00F0107001700548100400030000400015000500505014000430000000F80250076007280049030400075060090030000400060010600008 01491C004007100000
Mycobacterium leprae	00E1207001F12448100400030000400015000500505014000430000000F80250076007280049030400075060080050000400051110600008 01491F103007100000
Streptomyces bikiniensis	04A1207001911448100400030000400015000500505014000430000000FB02400760072800490304000650600800400004000600106000080 04A19214006100000
Streptomyces lividans 1326	04A1207001A11448100400030000400015001400515014000732000000CA13400760070A01410304000650600900400004000600106000080 04A0A204007100000
Clostridium acetobutylicum	06A1207001711348100400030000400015000500505014000430000000E8024007511725224903040106506000000000050005001060000800 4AA0000000100000
Bacillus brevis	25C0007001D0144810050000600600050500060040A11401540009663F800400E000000000030300A0000000000000000000000030700H800481D274008100000
Bacillus subtilis 168	47A0107001800548100500006106000504110600A070140157020A884C80040090000000000030300B00000000000000000000030700G600481D164007100000

Bacillus stearothermophilus	03E01070019004481005000061060005050006 00A0A0140154000B885F800400E000000000030300B0000000000000000000000030700F811481B385007100000
Mycoplasma capricolum	04B010700180064810050000600600050500 0600A070140134000C443E611400E000000000030300B0000000000000000000000030700H50048G0000000100000
Mycoplasma fermentans	007010700160044810050000600600050500060070201300043000000W000000000000000000030300B0000000000000000000000030600I600480400400600A114
Mycoplasma flocculare	0060107001M129481005000060060005050006007020130004300000 0EI7D600E000000000030300B0000000000000000000000030700HF0358A000000010B004
Mycoplasma genitalium	0070007001A00648100500006006000505000600A041270034000C554E700400E000000000030300B0000000000000000000000030700I50048C000000028E003
Mycoplasma hyopneumoniae	0070107001O0574810050000600600050500060070201400043000000EI7C500E000000000030300B0000000000000000000000030700GD1058A000000011B008
Mycoplasma pneumoniae	008000700160034810050000600600050500060090501801440 00B555D700400E000000000030300B0000000000000000000000030700H40058C000000027B006
Streptococcus pyogenes	05E0107001800448100500006006000504110600A070140154000A874F800400E000000000030300B00000000000000000000000030A00B600481C003007100000
Streptococcus pneumoniae	03E0107001900548100500006006000504110600A060140154000B774F800400E000000000030300B0000000000000000000000030800FC11281B004007100000
Streptococcus gordonii	32B0107001800548100500006006000504110600A070140154000A884F800400E000000000030300B0000000000000000000000030700H400481B005007100000
Staphylococcus aureus	08A0107001500348100500006006000504110600A030160154000 9994F800400E000000000030300A0000000000000000000000030700HA01581D385007100000
Enterococcus faecalis	25C0106111800448100500006006000504110600A070130154000A884F711400E000000000030300B0000000000000000000000030700H600481C004007100000
Bacillus megaterium	36B01070017004481005000060060005050006 00A0A01301540009A84F800400E000000000030300B0000000000000000000000030700G800481C274007100000
Rickettsia prowazekii	15823070016004481004000100007000150004007070140004300000 0F80230076007171149030400065080 0A0040000430030010600008004A06004007100000
Rhodobacter capsulatus	04B0107001D5554810040001000070001500050050501400043000000F802400760071800490304000650700734400002012400106000071 14A24004008100000
Agrobacterium tumefaciens	04B0107001C3044810040001000070001500050050501400043000000F902300760071800490304000650700800400002033300106000080 04A27006009100000
Rhodospirillum rubrum A348	04A1207001H4354810040001000070001500050050501400073200000C902500760070812420304010650700700400001022300206000080 04A0F134006100000
Caulobacter crescentus	0080807001900948100400010000700015000500505014000430000 00F813400760071800490304000650600600400001004600106000071 14A4911400B100000
Neisseria gonnorhoeae	04A1207001711A4810040003000040001500050050501400043000000GC23400760071801490304000550600000000003000411106000080 04AA0000000100000
Bordetella pertussis	00B0107001800448100400030000400015000500 50D454000430000 00EH22D42E000000000003040006506005004000020434002060000711 4A07004007100000
Ralstonia (*Alcaligenes*) *eutrophus* DSM531	04B0107001A1144810040003000040001500140051501400073200000BC1140090000000000030400065060000000000300050010600008004A1D004007100000
Neisseria menigitidis	04A1207001711A4810040003000040001500050050501400043000000GC23400760071801490304000550600000000003000411106000080 04AA0000000100000
Desulfovibrio desulfuricans	04B0107001711448100400030000400015000500505004000430000 00F902400760072800490304000650600000000000300500106000080 04A26004008100000
Helicobacter pylori J99	06900070015004481004000100006000150005005050040004300000 0F7023007600718004A030400077300000000000000000010600008004AB0000000100000
Helicobacter pylori 26695	06900070015004481004000100006000150005005050040004300000 0F6024007600718004A030400077300000000000000000010600008004AB0000000100000
Helicobacter pylori	06900070015004481004000100006000150005005050040004300000 0F7023007600718004A030400078300000000000000000010600008004AB0000000100000
Campylobacter jejuni	03A1106001500448100400030000500015000500505014000430000 00F61340075117262249030600040400000000000000000050600008004AB0000000100000
Chromatium vinosum	04B0107001800548100400030000400015000500505014000430000 00FB33400760071800490304000650600400600000012600106000080 04AA0000000100000
Thiobacillus (*AcidiThiobacillus*) *ferrooxidans g*	04B0107001611448100400030000400015000500505014000430000 00F933400760071800490304000650600000000000300050010600007114AA0000000100000
Erwinia agglomerulans	04B0107001I224481004000300004000240005005050160004300000 0ED23400760071801491304000650600000000000400050010600008004AA0000000100000
Thiobacillus ferrooxidans	04B0107001611448100400030000400015000500505014000430000 00F922400760071800490304000650600000000000300050010600008004AA0000000100000
Escherichia coli K12 W3110	04B0107001H224481004000300004000150005005040140006320000 0DD23400760070902430304000650600000000000400050010600008004AA0000000100000
Haemohilus influenza	04B0107001K674481004000300004000150005005040140004300000 0F70240076007180149030400065060000000000020005001060000 8004AA0000000100000
Klebsiella pneumoniae	04B0107001I224481004000300004000240005005050160004300000 0FD23400760071801491304000650600000000000400050010600008004AA0000000100000
Pseudomonas aeruginosa	04B0107001900448100400030000400015000500505014000430000 00E933400760071801490304000650600000000000300050010600008004AA0000000100000
Pseudomonas fluorescens	04B0107001A124481004000300004000150005005050140004300000 0E933400760071801490304000650600000000000200050010600007114AA0000000100000
Salmonella typhi	02B0107001G224481004000300004000150005005040150004300000 0FD23400760071801490304000650600000000000400050010600008004AA0000000100000
Salmonella typhimurium	04B0107001G224481004000300004000150005005040140004300000 0FD23400760071801490304000650600000000000400050010600008004AA0000000100000

Serratia marcescens	04B0107001G33448100400030000400015000500505014000430000000FD2340076007180149030400065060000000000040005001060000008004AA0000000100000
Shewanella putrefaciens	15A010700171144810040003000040001500050050501400043000000FC1340076007180149030400065060000000000040005001060000008004AA0000000100000
Yersinia pestis	02B0107001G334481004000300004000150005005050140004300000FD2340076007180149030400065060000000000040005001060000008004AA0000000100000
Treponema pallidum Nichols	0590027001700348100400010000600015000500 50A12400073200000C5024007511708014203040005505000000000003003002060000 8006AA0000000200000
Borrelia burgdorferi g	04B1106001A01448100400020000700015000500505003100440000000E5026007600718014903040006507000000000001300300206000080 04AB0000000100000
Borrelia burgdorferi	04B1106001A00448100400020000700015000500505003100440000000E5026007600718014903040006507000000000001300300206000080 04AB0000000100000
Thermotoga neapolitana	04B0007001901648100400010000400015000500505004000430000000G6025007600727004903040005505000000000001200400406000080 04AA0000000100000
Thermotoga maritima MSB8	04B0007001A01448100400010000400015000500505004000732000000D6025007600709014103040005505000000000001200400406000080 04AA0000000100000
Candidatus Kuenenia stuttgartiensis	05B0007001E24448100400030000400015000500505014000730000000CA02800790C409014203040006505000000000003600500106000080 04AA0000000100000

Archaea (30)

Metallosphaera sedula	20C0015003300448100400010000400016000500405005000731000000BJMD40070000000000003020001407000000000000000020070C0000000008000000200000
Sulfolobus acidocaldarius	13D0006003500348100400010000600015000400605004000731000000BPH740070000000000003020001407000000000003000300 20C000000000A0000000300000
Sulfolobus solfataricus	32B0015003500448100400010000600015000500405005000731000000ANED4007000000000000302000350600000000000200030020C0000000009000000300000
Aeropyrum pernix KT	00K0006003900548100400010000600015000500406003000731000000BNHG4007000000000000302000040900000000000310030010C000000000B0000000200000
Archaeoglobus fulgidus	14D1206003200448100400050000000004004000006004000901000000CFB640080000000000003030040000000000000000000000C0000000009000000100000
Methanococcus jannaschii JAL 1	117000600370034810050006000000000700100000610400 0A0000000CGC640080000000000003040040000000000000000000000C000000000C0000000200000
Methanococcus vannielii	00C110600320054810040004000000000500400000500410 0A0010000BIB530070000000000003030030000000000000000000000C000000000C0000000200000
Methanococcus maripaludis	00C110600320044810050004000000000500300000500410 0A0000000BJA530070000000000003020040000000000000000000000C000000000C0000000200000
Methanococcus thermolithotrophicus	00B110600320054810040003000000000411600000500410 0A0010000BG66B0070000000000003020040000000000000000000000C000000000C0000000200000
Halobacterium cutirubrum	05A1206004600448100400010000800024000600405004000632000000CcEEE47800000000000139000000000003004000090220000 0C000000000A0000000100000
Methanobacterium formicicum	00F1206003E02548100400010000500025000500405004000631000000CH944008000000000000304000550500000000000300030020C000000000A0000000100000
Halococcus morrhuae	04A1206003G45448100400010000600015000500505004000632000000C$KOC6C8000000000000390010000000400500002062000000C000000000A0000000100000
Methanobacterium thermoautotrophicum ΔH g	05F1206003700448100400010000500025000500405004000631000000CHC78008000000000000304000550500000000000300030020C0000000009000000100000
Methanobacterium thermoautotrophicum ΔH 1	11701060037004481004000100005000250005004050040006310000000CHC98008000000000000304000550500000000000300030020C0000000009000000100000
Methanobacterium thermoautotrophicum Marburg	00F1206003700448100400010000500025000500405004000631000000CHC99008000000000000304000550600000000000300020020C0000000009000000100000
Natronobacterium gregoryi	04A1206003911448100400010000800015000511405004000632000000C+EHJ6H80000000000003B000000000070030000430000000C000000000A0000000100000
Halobacterium sp. NRC1	05A1206003600448100400010000800024000600405004000632000000CU6BE4Q800000000000139000000000003004000090220000 0C000000000A0000000100000
Haloferax volcanii DS 2	04A1206003800348100400010000700015000600405004000632000000Cm66D6Q80000000000003900000000007006A114A01200000C000000000A0000000100000
ER E wastewater enrichment	00F1206003E02548100400010000500025000400405004000631000000CJA78008000000000000304000550000000000000300020020C0000000008000000100000
Halobactrium trapanicum	46B1206003H13648100400010000600015000600305003000632000000CRDG400800000000000039001000000050040000306200000C000000000A0000000100000
ER H wastewater sludge	00F1206003E02548100400010000500025000500405004000631000000CH965008000000000000304000550500000000000300030020C000000000A0000000100000
Methanosarcina barkeri	14E1206003600448100400020000600025000600505004000531000000EH75400A000000000000303000390700000000000100300 80C0000000000N127337100000
Pyrococcus furiosus 1	00E0006003500548100400010000700015000500405004000631000000CNIH400800000000000030400066050000000000030003002 0C000000000B0000000200000
Thermococcus celer AL 1	00E0006003400648100400010000700015000500405004000531000000BNIH400600000000000030400066050000000000030003002 0C000000000C0000000200000
Pyrococcus horikoshii OT3	00E0006003500348100400010000700015000500405004000731000000BNIH400600000000000030400066050000000000030003002 0C000000000B0000000200000
Thermoplasma acidophilum	01G0106003400548100400010000600015000600405004000631000000CF99300700000000000030400055050000000000030003001 0C0000000005003007100000
Thermococcus litoralis	0170005003500548100400010000700015000500405004000631000000CNIH400800000000000030400066050000000000030003002 0C000000000C0000000200000
Pyrococcus abyssi	00D1106003400648100400010000700015000500405004000731000000BNIH400700000000000030400066050000000000030003002 0C000000000B0000000200000

Pyrococcus furiosus 2	00E000600350044810040001000070001500050040500400073100000BNIH400700000000000030400066050000000000300030020C000000000C0000000200000
Thermoplasma volvanum	01G010600340044810040001000060001500060040500400063100000CF993007000000000000304000550500000000000200030020C00000000005004007100000
**Eukarya (26)**	
Reclinomonas americana (mitochondria)	00B010700160034810040006000000000500050050501400043000000E800B00740030800660304004000000000000000000000000060000500CA0B004006100000
Porphyra purpurea (chloroplast)	04532070018016481004000200007000151004007050140007320000BA44300760070901412303110680600000000000110020040600008004A1A224007100000
Zea mays (chloroplast)	68F3315000612A1910340000000070003501071240521400043000000B602200700000B03481302000670500000000000130040030300005003912004003300000
Cyanophora paradoxa (cyanelle)	04B000700160054810050001000070001510050050501400043000000EA023007511736004A03030005F0600000000000110020020600008004AC0000000200000
Pongo pygmaeus	01C103400599B868005000000000000000400660030800300060000000ES33B3D90000000000K00000000000000000000000000000000000000000A105007400000
Mus musculus	00D0024005995568005000000000000000400650050600400060000000EM579369000000000K000000000000000000000000000000000000000009104007400000
Homo sapiens	01C103400599B858105000000000000000400650050700500070000000DV56A338000000000K000000000000000000000000000000000000000000B103007500000
Gorilla gorilla	00C103400599B868005000000000000000400640070700500060000000ES33A3D90000000000K00000000000000000000000000000000000000000A105007200000
Macaca mulatta	01C103400599B868005000000000000000400660030700500060000000ES33B3D90000000000K00000000000000000000000000000000000000000A105007400000
Danio rerio	3291125004997568005000000000000000400650050500500060000000EQ34F348000000000I000000000000000000000000000000000000000007003007400000
Pan troglodytes	01C103400599B868005000000000000000400640070700500060000000ES33A3D90000000000K00000000000000000000000000000000000000000A105007400000
Schizosaccharomyces versatilis	02L0026002AAA485000300020000000000500M000005005300600200000CB0170080000000000000E0000000000000000000000000000000000000005005007700000
Saccharomyces diastaticus	12D0026009DAC485000400050000000000500AC004D860480060040000CB00400800000000000000C23600000000000000000000000E00000000005004007500000
Saccharomyces uvarum	12D0026009DAC485000400050000000000500AC004D860480060040000CB00400800000000000000C23600000000000000000000000E00000000005004007500000
Saccharomyces kluveri	12C1116005EBB485000400050000000000500C6004F800740060040000C6004007000000000000000B21400000000000000000000000E00000000005006007500000
Schizosaccharomyces japonicus	02L0026002AAA485000300020000000000500M000005004300600200000CB0170080000000000000E0000000000000000000000000000000000000005005007700000
Schizosaccharomyces malidevorans	02L0026002AAA485000300020000000000500M000005004300600200000CB0170080000000000000E0000000000000000000000000000000000000005005007700000
Schizosaccharomyces octosporus	13K0026002BAA485000300020000000000500M000005004300600200000C500E008000000000000E0000000000000000000000000000000000000005008007700000
Saccharomyces globosus	12C11260079AD385000700050000000000500Q00000611481060006224D9003008000000000000000912500000000000000000000000E00000000006114007500000
Saccharomyces bayanus	12D0026009DAC485000400050000000000500AC004D860480060040000CB00400800000000000000C23600000000000000000000000E00000000005004007500000
Tarsius syrichta	01C103400599B868005000000000000000400730030D700500051100000ES34B3D90000000000G00000000000000000000000000000000000000000A107007400000
Saccharomyces carlsbergensis	12C1126009CAB485000400050000000000500AB003D860480060040000DA00500800000000000000C23400000000000000000000000E00000000005004007500000
Saccharomyces cerevisiae	12D0026009DAC485000400050000000000500AC004D860480060040000CB00400800000000000000C23600000000000000000000000E00000000005004007500000
Xenopus laevis	03E00150039AB858005000000000000000300750050600500060100000DPI47008000000000000K000000000000000000000000000000000000000002008008200000
Schizosaccharomyces pombe	02L0026002AAA485000300020000000000500M000005004300600200000CB0170080000000000000E0000000000000000000000000000000000000005005007700000
Entamoeba histolytica	0090037000F02938100400000000000000300550031600400061010000BD32500700000000001G00000000000000000000000000000000000000005003008400000
